# Point mutations in functionally diverse genes are associated with increased natural DNA transformation in multidrug resistant *Streptococcus pneumoniae*

**DOI:** 10.1093/nar/gkae1140

**Published:** 2024-12-03

**Authors:** Flora Peillard-Fiorente, Nguyen Phuong Pham, Hélène Gingras, Chantal Godin, Jie Feng, Philippe Leprohon, Marc Ouellette

**Affiliations:** Centre de Recherche en Infectiologie, Axe des Maladies Infectieuses et Immunitaires du CHU de Québec and Département de Microbiologie, Infectiologie et Immunologie, Faculté de Médecine, Université Laval, 2707 Bd Laurier, Québec, QC G1V 4G2, Canada; Centre de Recherche en Infectiologie, Axe des Maladies Infectieuses et Immunitaires du CHU de Québec and Département de Microbiologie, Infectiologie et Immunologie, Faculté de Médecine, Université Laval, 2707 Bd Laurier, Québec, QC G1V 4G2, Canada; Centre de Recherche en Infectiologie, Axe des Maladies Infectieuses et Immunitaires du CHU de Québec and Département de Microbiologie, Infectiologie et Immunologie, Faculté de Médecine, Université Laval, 2707 Bd Laurier, Québec, QC G1V 4G2, Canada; Centre de Recherche en Infectiologie, Axe des Maladies Infectieuses et Immunitaires du CHU de Québec and Département de Microbiologie, Infectiologie et Immunologie, Faculté de Médecine, Université Laval, 2707 Bd Laurier, Québec, QC G1V 4G2, Canada; State Key Laboratory of Microbial Resources, Institute of Microbiology, Chinese Academy of Sciences, 52, Sanlihe Rd, Xicheng District, Beijing 100045, China; Centre de Recherche en Infectiologie, Axe des Maladies Infectieuses et Immunitaires du CHU de Québec and Département de Microbiologie, Infectiologie et Immunologie, Faculté de Médecine, Université Laval, 2707 Bd Laurier, Québec, QC G1V 4G2, Canada; Centre de Recherche en Infectiologie, Axe des Maladies Infectieuses et Immunitaires du CHU de Québec and Département de Microbiologie, Infectiologie et Immunologie, Faculté de Médecine, Université Laval, 2707 Bd Laurier, Québec, QC G1V 4G2, Canada

## Abstract

DNA transformation is key for phenotypic diversity and adaptation of *Streptococcus pneumoniae* including in the emergence of multidrug resistance (MDR). Under laboratory conditions, DNA transformation is facilitated by the artificial triggering of competence by the competence stimulating peptide (CSP). In ongoing DNA transformation work, we observed that exogenous CSP was dispensable depending on the combination of strains and culture media. Here, we carried out a chemogenomic screen to select for *S. pneumoniae* mutants capable of natural transformation in medium that normally would not sustain natural transformation. Our chemogenomic screen relied on chemical mutagenesis followed by selection of mutants with increased DNA transformation capacities. Sequencing the genome of these mutants revealed an abundance and diversity of mutated genes proven experimentally to increase natural transformation. A genome wide association study between MDR and sensitive clinical isolates revealed gene mutations associated with MDR, many of which intersected with those pinpointed by our chemogenomic screens and that were proven to increase natural transformation. *S. pneumoniae* has adopted DNA transformation as its lifestyle and can select for mutations facilitating DNA transformation.

## Graphical Abstract

**Graphical Abstract:**
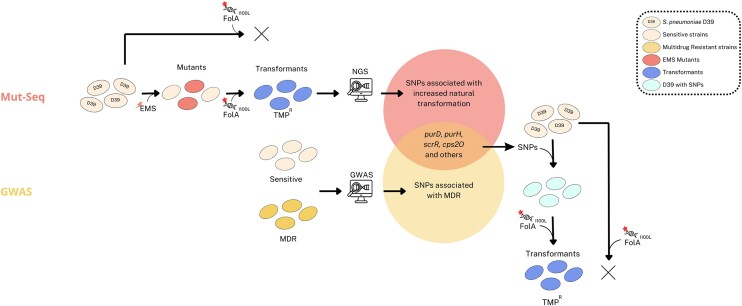

## Introduction

*Streptococcus pneumoniae* colonizes asymptomatically the human nasopharynx but is also a successful pathogen. Despite the availability of conjugated vaccines for the most common serotypes, it remains responsible for severe diseases and is associated with approximately 300 000 deaths annually in children (1,2). Natural genetic transformation allows this pathogen to uptake exogenous DNA for recombination into its genome, leading to increased phenotypic diversity including antimicrobial resistance (AMR), virulence or vaccine escape [reviewed in Salvadori *et al.* (3)]. Studies of natural transformation following the induction of a competence state in *S. pneumoniae* have a long and rich history culminating in showing that DNA was the genetic material (4,5).

Competence, necessary for DNA transformation, was known to be restricted to a small time window at a defined cell density (6). The discovery of the competence stimulating peptide (CSP) (7) led to major advances in our understanding of competence and in its exploitation for DNA transformation work. This is especially true with encapsulated *S. pneumoniae* clinical isolates for which natural transformation is hard to achieve *in vitro* (8,9). The addition of exogenous CSP is now standard for the artificial triggering of the competence state in *S. pneumoniae*.

While natural transformation, i.e. without exogenous CSP supplementation, is inefficient *in vitro*, this may differ *in vivo* as the competence state of *S. pneumoniae* is prolonged in animal model of *S. pneumoniae* sepsis (10) and natural transformation was shown to occur in biofilms of nasopharyngeal carriage at even a higher rate (9). Biofilms in the nasopharynx are known to induce the expression of competence genes (11) and the use of biofilms *in vitro* has permitted natural transformation in *S. pneumoniae* isolates refractory to natural transformation when grown under planktonic conditions (9). The enhanced expression of competence genes in biofilms is probably due to several factors including the high cell density activating the CSP-linked quorum sensing (12); the stresses induced under nutrient-limited conditions (13); and the presence of extracellular DNA in the biofilm’s extracellular matrix (13).

We extensively used genome transformation in *S. pneumoniae*, made competent by supplementation with CSP, to reconstruct AMR phenotypes from resistant isolates (8,14–16). In efforts to increase the efficiency of transformation, we moved to biofilms and confirmed as others (9) that exogenous CSP was dispensable but, as described herein later, only in certain growth medium. Intrigued by this medium-dependent natural transformation, we designed a chemogenomic screen selecting for increased natural transformation in media normally not supporting transformation. This screen pointed at an abundant and diverse gene pool contributing to natural DNA transformation. We hypothesized that *S. pneumoniae* multidrug resistant (MDR) isolates, which presumably went through multiple rounds of transformation, may also have been selected for mutations favoring natural transformation. Genome wide association studies (GWAS) supported this hypothesis and revealed a diversity and abundance of nucleotide variants associated with MDR isolates. Some of the variants were in genes common to both the chemogenomic screens and GWAS. DNA transformation is a way of life for *S. pneumoniae*, and it is possible that clinical isolates have accumulated mutations that can increase natural transformation to adapt to new challenges.

## Materials and methods

### Bacterial strains, plasmids and growth conditions

*S. pneumoniae* strains used in this study are listed in Supplementary Table S1. Bacteria were grown at 35°C with 5% CO_2_ on Trypticase soy agar with 5% sheep blood (TSAII, Becton Dickinson), in brain heart infusion (BHI, Becton Dickinson), Columbia (Nutri-bact), semi-synthetic medium C + Y (6) or in the synthetic medium CDM (17,18). Minimal inhibitory concentrations (MICs) were determined as previously described (19).

### Biofilm formation

*S. pneumoniae* D39, TIGR4, ATCC-49647 and CCRI-14647 (Supplementary Table S1A) were grown in CDM, C + Y, BHI or Columbia to an OD_600_ of 0.1 equivalent to 4 × 10^7^ colony-forming units per milliliter (CFU/ml), at which point 1 ml of culture was diluted as 1:2 with fresh medium and seeded to the bottom of a sterile 24-well plate and incubated at 35°C under a 5% CO_2_ atmosphere. Culture supernatant was carefully changed every 24 h to avoid autolysis. After 48 h (for the nutrient-rich media BHI and Columbia) or 72 h (for the semi-chemically defined medium C + Y and the synthetic medium CDM) of biofilm growth, biofilms were washed thrice with 1 × phosphate-buffered saline (PBS). The plates were then sealed and sonicated for 3 s (by floating in a water bath sonicator) to disperse bacteria. Cells from the dispersed biofilm were collected and the number of viable CFU per ml was determined by plating on TSAII; a value referred to as the total CFU per biofilm (17).

### Transformation of mutations from candidate genes

Polymerase chain reaction (PCR) fragments of 5 kb covering the mutations (2.5 kb flanking the mutation) from the genes of interest derived from the chemical mutagenesis (Mut-Seq) screen were generated with the Phusion enzyme (Thermo Scientific) using primers listed in Supplementary Table S2. For GWAS-derived mutations, the 5 kb PCR fragments used for transformation were generated by a PCR fusion strategy with primers harboring the mutations (Supplementary Table S2). The mutated PCR products were co-transformed in competent *S. pneumoniae* D39 cells along with a PCR fragment (500 bp) of the *rpsL^+^* allele conferring streptomycin (STR) resistance, as described (14). The *rpsL^+^* allele codes for the RpsL^K56T^ mutation (20) and was amplified from *S. pneumoniae* CP1296. The transformants were selected on CAT agar plates supplemented with 5% (vol/vol) sheep blood containing STR (150 μg/ml) for selection. The insertion of the co-transformed mutations was validated by Sanger sequencing. The PCR fragment covering the *folA*^+^ allele coding for the FolA^I100L^ mutation conferring resistance to trimethoprim (TMP) was amplified from *S. pneumoniae* R6 FolA^I100L^ (19). TMP at 16 μg/ml was used for selection of transformants on CAT agar plates supplemented with 5% (vol/vol) sheep blood.

### Natural transformation efficiency assays

Cell lysates from *S. pneumoniae* D39-RpsL^K56T^ or FolA^I100L^ were generated from culture grown to an OD_600_ of 0.4 and heated at 95°C for 5 min. The absence of viable cells from the lysates was confirmed by plating on TSAII (9). For biofilm natural transformation, *S. pneumoniae* D39, TIGR4, CCRI-14647 or ATCC-49647 biofilms were grown in CDM, C + Y, BHI or Columbia medium for 24 h at 35°C under a 5% CO_2_ atmosphere, after which the culture supernatant was carefully removed and changed for a 1:25 mixture of either *S. pneumoniae* D39-RpsL^K56T^ or D39-FolA^I100L^ lysates diluted in fresh medium. The pH of the various media remained constant at 8 throughout those experiments. Biofilms were then incubated at 35°C in 5% CO_2_ for 48 h (BHI and Columbia) or 72 h (C + Y and CDM); the supernatant being carefully changed every 24 h to avoid autolysis. The biofilms were harvested and plated on TSAII to calculate the total CFU per biofilm and on CAT agar with 5% (vol/vol) sheep blood plates supplemented with STR (150 μg/ml) or TMP (16 μg/ml) to assess the number of transformants. For planktonic natural transformation, *S. pneumoniae* was grown at 35°C under a 5% CO_2_ atmosphere until the OD_600_ reached an equivalent of 4 × 10^7^ CFU/ml (OD_600_ = 0.1), at which point 1 ml of culture was diluted 1:2 with 1 ml of fresh medium, seeded in a 24-well plate and supplemented with 1:25 lysate of *S. pneumoniae* D39-RpsL^K56T^ or D39-FolA^I100L^. The culture was grown for 4 h, before biofilms could form (21), and plated on TSAII to calculate the total CFU and on CAT agar containing antibiotics to assess the number of transformants. Transformation efficiency was calculated as the number of transformants obtained over the total number of pneumococcal cells recovered.

### Mut-Seq screens selecting for increased natural transformation

The chemical mutagenesis of *S. pneumoniae* was performed as previously described (19). A colony of *S. pneumoniae* D39 was grown in 100 ml of BHI to an OD_600_ of 0.1 equivalent to 4 × 10^7^ CFU/ml. The culture was split into 10 ml cultures and incubated at 35°C with 8× or 16× the MIC of ethyl methanesulfonate (EMS; Sigma–Aldrich). Nonmutagenized control cells were processed similarly. After 20 min of incubation, 10 ml of cold BHI medium was added, and the culture was diluted 1/10 in BHI and incubated for 3 h for cell recovery. Bacteria were harvested by centrifugation (10 min at 3220 × *g*) and resuspended in C + Y medium. These cells were inoculated into a 24-well plate for biofilm formation and exposed to a DNA lysate derived from D39-FolA^I100L^. After 72 h of incubation at 35°C (changing supernatant every 24 h to avoid autolysis), the biofilm was harvested and 150 μl were spread on CAT agar with 5% (vol/vol) sheep blood containing 16 μg/ml of TMP. Total CFU counts were determined by serial 1:10 dilutions and spread on TSAII. The agar plates were incubated overnight at 35°C. TMP-resistant clones were picked from the CAT agar plates and subjected to a secondary screening, this time with a *S. pneumoniae* D39-RpsL^K56T^ lysate. This secondary screening was required to ensure that we worked with clones that had acquired TMP-resistance due to a genuine increase in transformation and not from *de novo* selection of TMP-resistance from the chemical mutagenesis. Clones resistant to TMP and STR were recovered from CAT agar with 5% (vol/vol) sheep blood supplemented with 16 μg/ml of TMP and 150 μg/ml of STR.

### Genome sequencing

Genomic DNA (gDNA) was extracted using the Wizard Genomic DNA purification kit (Promega) according to the manufacturer’s protocol. The purity of the gDNA was assessed using a NanoDrop spectrophotometer and its quantification was done using a QuantiFluor One double-stranded DNA system (Promega). Illumina Nextera XT sequencing libraries were prepared from 0.8 ng of gDNA according to the manufacturer’s instructions. The size distribution of the Nextera XT libraries was validated using a 2100 Bioanalyzer and high-sensitivity DNA chips (Agilent Technologies). Sequencing was performed on an Illumina MiSeq (paired-ends 150 nucleotide reads) for the Mut-Seq in C + Y medium.

### Single-nucleotide polymorphism and Indel detection in Mut-Seq mutants

Next-generation sequencing reads were aligned to the *S. pneumoniae* D39V genome using the software BWA-MEM (22) (with the -M option for Picard compatibility). The maximum number of mismatched was four, the seed length was 32 and two mismatches were allowed within the seed. Read duplicates were marked using Picard (http://broadinstitute.github.io/picard), and we applied GATK for single-nucleotide polymorphism (SNP) and Indels discovery (23) using default settings.

### Genome wide association studies

A first pilot GWAS (GWAS-1) was conducted using 108 multiresistant *S. pneumoniae* strains and 108 susceptible strains derived from three studies (8,24,25) (Supplementary Table S1B). For a second in-depth GWAS (GWAS-2), metadata from the Global Pneumococcal Sequencing project was retrieved from the Monocle database (https://data-viewer.monocle.sanger.ac.uk/project/gps, accessed on 12 January 2024). The MIC values were interpreted according to Clinical & Laboratory Standards Institute guidelines (26), and isolates showing resistance to at least three of the six antibiotics (benzylpenicillin, ceftriaxone, cotrimoxazole, erythromycin, tetracycline and chloramphenicol) were considered MDR. Metadata analysis showed that in many Global Pneumococcal Sequence Clusters (GPSCs), MDR strains are more prevalent than susceptible strains, and *vice versa* (Supplementary Table S3). We then selected 425 MDR and 425 susceptible *S. pneumoniae* strains from 14 GPSCs, with the number of MDR and susceptible strains selected in each GPSC broadly reflecting their prevalence, as input for GWAS-2 (Supplementary Table S1C). SNPs were called by Snippy (version 4.6.0; https://github.com/tseemann/snippy), using the *S. pneumoniae* D39V genome as reference (27) (parameters: –minqual 100 –mincov 10 –minfrac 0.9). Gubbins (version 3.3.1) (28) was used to detect recombinant regions and generate phylogenetic tree (parameters: –tree_builder fasttree –sh-test –iterations 10). Associations of nonsynonymous SNPs in coding regions with the MDR phenotype were investigated using Pyseer (version 1.3.11) (29) with a linear fixed effects model (Sequence element enrichment analysis (SEER)) with multidimensional scaling of genetic distances to correct for population structure. Multiple testing correction was performed using a Bonferroni correction (α = 0.05) with the number of unique SNP patterns as the number of multiple tests. Pyseer’s *P*-value thresholds for significance after Bonferroni correction were 2.43 × 10^−6^ for GWAS-1 and 1.85 × 10^−6^ for GWAS-2. An SNP was classified as associated with MDR if it (i) had a significant *P*-value of association after Bonferroni correction, (ii) was present in MDR strains at a ratio of ≥5 compared with susceptible strains and (iii) was present in at least 30 more MDR strains than susceptible strains. In GWAS-1, we used Pyseer’s filter *P*-value (unadjusted for population structure), while in GWAS-2, we used Pyseer’s lrt *P*-value (adjusted for population structure).

## Results

### Natural DNA transformation is dependent on culture medium and genetic background

*S. pneumoniae* D39 biofilms were grown in the chemically defined C + Y (6) or CDM (17) media. Biofilms had a biomass ranging from 1 × 10^7^ to 3 × 10^8^ CFU/ml, a value in line with mature biofilm formation in *S. pneumoniae* (17). The total viable CFU counts per biofilm was compared with those of planktonic cultures after the addition of 500 mg/ml of gentamicin for 3 h at 35°C. Tolerance to gentamicin is a known property of biofilms (17). Gentamicin caused a 4-log reduced viability of the planktonic cultures, but not of our biofilms. Lastly, the quality of our biofilms was evaluated by measuring the expression of *comD* and *licD2* by quantitative reverse transcriptase PCR (qRT-PCR), upregulated in biofilms by 10- and 3-fold compared with planktonic cells, respectively (Supplementary Figure S1), consistent with published work (30).

**Figure 1.**
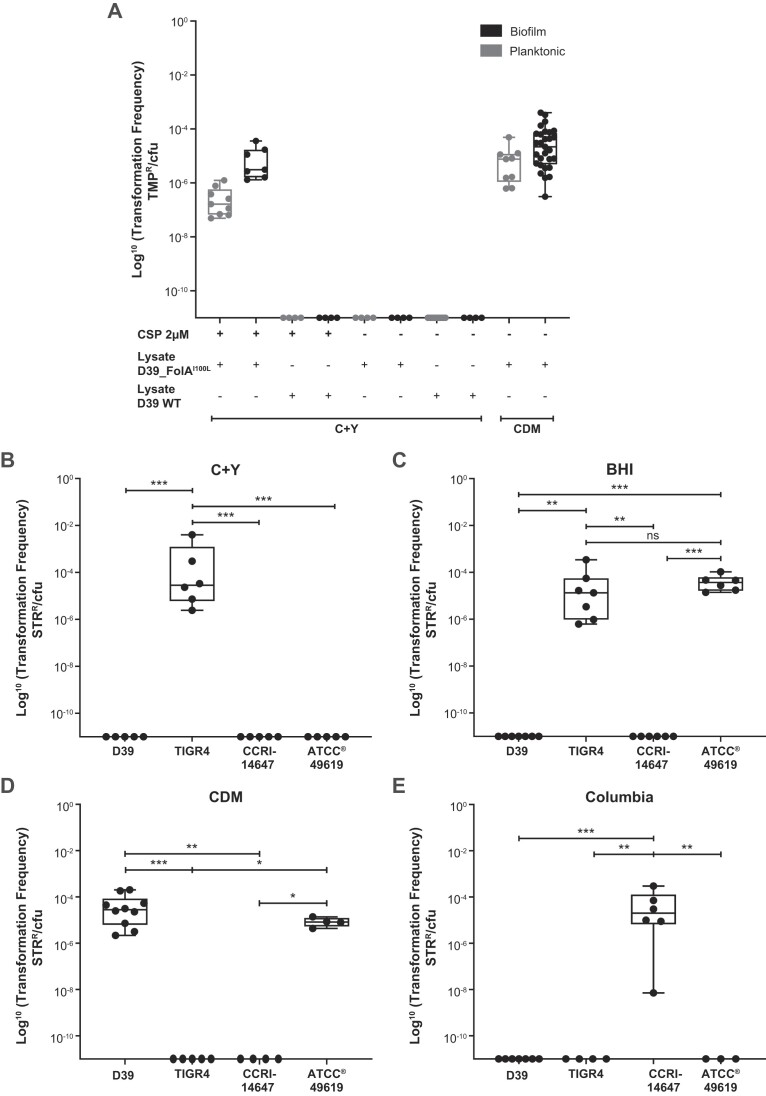
DNA transformation in *S. pneumoniae* depends on environment and genetic background. (**A**) The transformation efficiency of *S. pneumoniae* D39 grown as biofilm or planktonic cells was monitored in C + Y and CDM media following exposure to a D39-FolA^I100L^ lysate or D39 WT lysate. The acquisition of the mutation leading to the FolA^I100L^ substitution confers TMP resistance to the transformants. For the C + Y medium, we compared transformation efficiency in the presence (+) and absence (−) of exogenous CSP-1. (**B**–**E**) The natural transformation efficiency of *S. pneumoniae* D39, TIGR4, ATCC 49619 and CCRI-14647 grown as biofilms was monitored in media C + Y, BHI, CDM or Columbia following exposure to a D39-RpsL^K56T^ lysate. Transformation efficiency is reported as the number of antibiotic-resistant transformants over the number of viable cells. Each point represents a biological replicate. Transformation efficiencies were compared using the Kruskal–Wallis test followed by Dunn’s test. Significance is indicated as follows: *P < 0.05; **P < 0.01; ***P < 0.001.

For the transformation efficiency assay, biofilms grown for 24 h were exposed to a cell lysate derived from *S. pneumoniae* D39 harboring the FolA^I100L^ allele which confers resistance to TMP and incubated for 72 h. This D39-FolA^I100L^ lysate was also used with planktonic *S. pneumoniae* D39. Transformation efficiency was determined from cell counts obtained by plating cells in the presence and absence of TMP. In C+Y medium, transformation was observed only when exogenous CSP-1 was added, and it was more efficient in biofilms than in planktonic cells (Figure 1A). This was not the case when cells were grown in CDM, transformants being observed in this medium even in the absence of exogenous CSP-1 and at a transformation efficiency similar between planktonic and biofilm conditions (Figure 1A). To extend this observation, we tested the ability of four strains (D39, TIGR4, ATCC 49619 and CCRI-14647) to transform a RpsL^K56T^ mutation when exposed to a lysate of *S. pneumoniae* D39 coding for this S12 ribosomal protein variant, which confers resistance to STR, in four media in the absence of exogenous CSP. This led to diverse transformation profiles, each strain being unique as for the media allowing natural transformation (Figure 1B–E). TIGR4 and ATCC 49619 could both transform in BHI (Figure 1C) but TIGR4 also transformed in C + Y (Figure 1B). ATCC 49619 could transform also in CDM (Figure 1D), the only medium in which D39 showed natural transformation (Figure 1A and D). The Columbia medium supported natural transformation only with strain CCRI-14647 (Figure 1E).

### Mut-Seq screening enables selection of mutants with increased natural transformation

**Figure 2.**
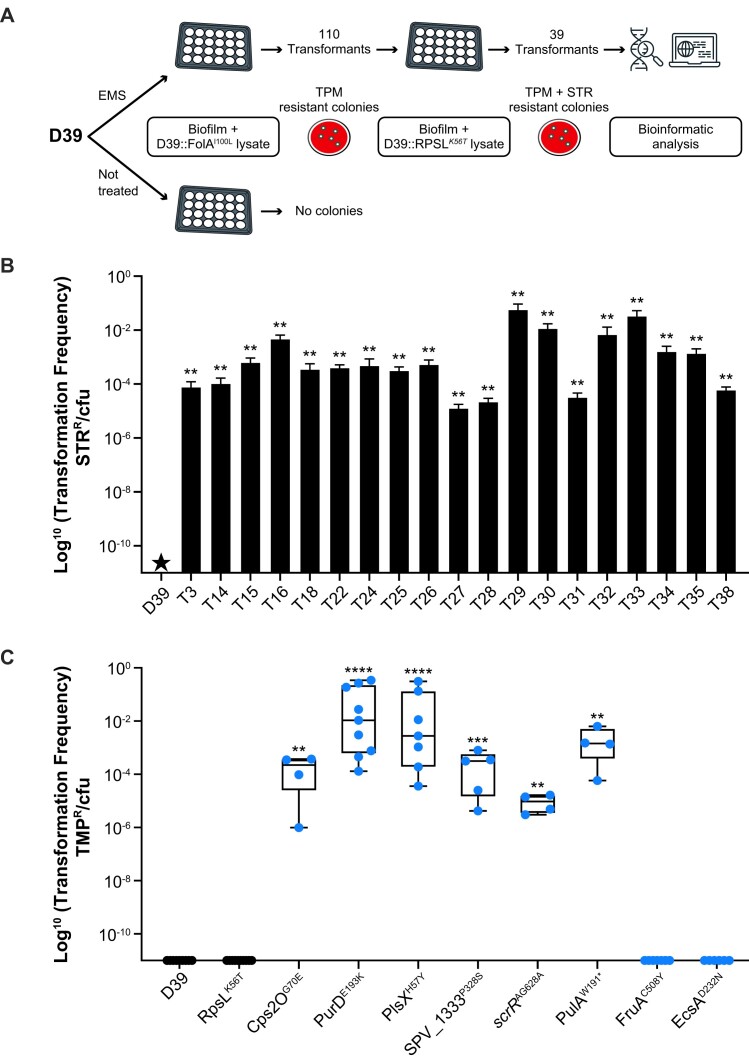
Selection for increased transformation efficacy in *S. pneumoniae* D39 using Mut-Seq. (**A**) Overview of the Mut-Seq screen. *S. pneumoniae* D39 mutagenized with EMS and grown as biofilm in C + Y medium in 24-well plates were exposed to a cell lysate derived from *S. pneumoniae* D39 coding for the FolA^I100L^ variant, which confers resistance to TMP. Transformants were selected on TMP-containing agar plates and grown again as biofilms in C + Y medium in 24-well plates. No TMP-resistant transformants were obtained from nonmutagenized control cells processed in parallel. To ascertain that the TMP-resistant phenotype results from increased transformation efficiency and not directly from mutagenesis, 110 randomly picked TMP-resistant transformants were subjected to a second round of transformation using a cell lysate derived from *S. pneumoniae* D39 coding for the RpsL^K56T^ variant, which confers resistance to STR. This led to 39 transformants resistant to both TMP and STR, whose genomes were sequenced. Of these, 19 had acquired the expected FolA^I100L^ at the first round of transformation and were as such considered to have genuine increased natural transformation capacities. (**B**) The increased natural transformation efficiency of the 19 *S. pneumoniae* D39 FolA^I100L^-positive transformants derived from the Mut-Seq screen was further assessed as biofilm in C + Y following exposure to a D39-RpsL^K56T^ lysate. Transformation efficiency is reported as the number of STR-resistant transformants over the number of viable cells. Error bars represent the standard error calculated from biological replicates (*n* = 6). Transformation efficiencies were compared with the one of D39 wild type (WT) using the Kruskal–Wallis test followed by Dunn’s test. **P < 0.01. (**C**) A selection of six mutations detected by Mut-Seq in at least two independent mutants (see Table 1) along with two mutations not expected to increase natural transformation were introduced in the genome of *S. pneumoniae* D39. The natural transformation efficiency of the transformants was monitored with cells grown as biofilms in C + Y following exposure to a cell lysate derived from *S. pneumoniae* D39 coding for the FolA^I100L^ variant. Transformation efficiency is reported as the number of TMP-resistant cells over the number of viable cells. Each point represents a biological replicate. Transformation efficiencies were compared with the one of D39 using the Kruskal–Wallis test followed by Dunn’s test. *P < 0.05; **P < 0.01; ***P < 0.001; ****P < 0.0001.

To investigate the basis of natural transformation, we adapted a chemogenomic screen (Mut-Seq) (31) using EMS, selecting for *S. pneumoniae* D39 capable of transformation in C + Y medium in the absence of exogenous CSP-1 (Figure 2A). The mutagenized bacteria, grown as biofilms in C + Y medium, were incubated with the D39-FolA^I100L^ lysate and upon plating of the biofilm, mutagenized cells, but not the nonmutagenized control, led to TMP-resistant colonies (Supplementary Figure S2). We randomly picked 110 mutated TMP-resistant transformants and grew them as biofilms in C + Y medium. These transformants were submitted to a second round of transformation but this time with the D39-RpsL^K56T^ lysate and plated on agar supplemented with both TMP and STR. This secondary screening was performed to increase the likelihood that TMP-resistant transformants acquired TMP-resistance from genuine increased transformation rather than from *de novo* selection of TMP-resistance from the chemical mutagenesis. Thirty-nine transformants grew on plates containing both antibiotics (Figure 2A). We sequenced the genomes of these 39 transformants, along with 11 TMP-resistant clones that did not lead to STR-resistant colonies after secondary screening (negative transformation clones). A total of 166 SNPs and Indels [126 in 57 coding sequences (Supplementary Table S4) and 40 in 13 intergenic regions (Supplementary Table S5)], were identified in the 39 clones resistant to TMP and absent in the 11 negative transformation clones. Only 19 of these 39 transformants had the expected FolA^I100L^ genotype after the first round of screening (Supplementary Table S4). The genomes of the 20 remaining TMP-resistant mutants (Supplementary Figure S3A) instead harbored mutations either in the *folA/dpr* intergenic regions disrupting a predicted rho-independent terminator upstream of *folA*, or coding for the FolA^L31F^ variant (Supplementary Table S5), both proven to confer TMP resistance (19). We excluded these 20 mutants from further analysis and focused on the 19 transformants with the FolA^I100L^ genotype, whose natural transformation efficiency was confirmed to be enhanced and ranging from 1 × 10^−5^ up to 1 × 10^−1^ when grown as biofilms (Figure 2B). We are confident that for these cells the FolA^I100L^ genotype has arisen from a genuine transformation event rather than being the product of EMS mutagenesis, as EMS induces transition (19) while a T298G transversion is required for a I100L mutation. Interestingly, this increased capacity of natural transformation was also observed in 14 of the 19 FolA^I100L^ transformants when assayed as planktonic *S. pneumoniae* (Supplementary Figure S3B).

### Mutations identified in the Mut-Seq screens increase natural transformation efficiency

**Figure 3.**
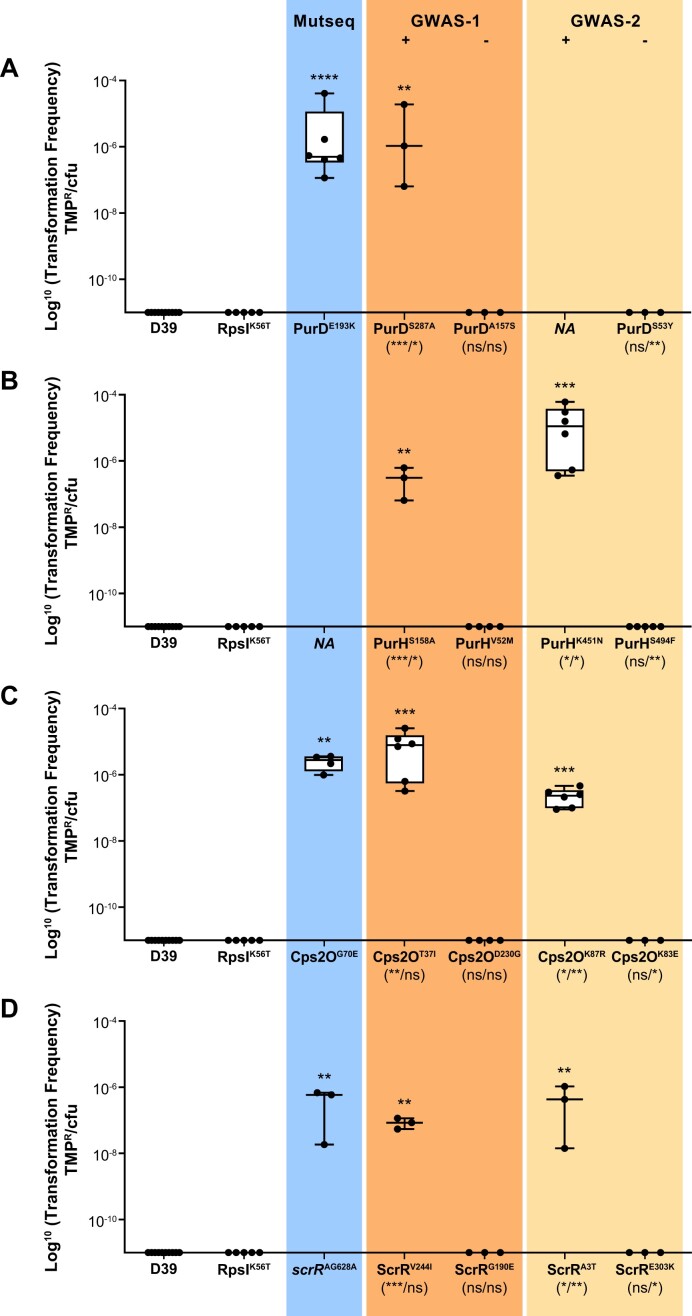
Mutations in diverse genes intersecting with Mut-Seq and GWAS screens increase natural transformation in *S. pneumoniae*. Mutations detected in PurD (**A**), PurH (**B**), Cps2O (**C**) and ScrR (**D**) by Mut-Seq (blue) or in the two GWAS screens (tan, GWAS-1; beige, GWAS-2) were introduced in *S. pneumoniae* D39. The natural transformation efficiency of the transformants was monitored with planktonic cells grown in C + Y media following exposure to a cell lysate derived from *S. pneumoniae* D39 coding for the FolA^I100L^ variant. Transformation efficiency is reported as the number of TMP-resistant cells over the number of viable cells. Each point represents a biological replicate. Transformation efficiencies were compared with those of D39 using the Kruskal–Wallis test followed by Dunn’s test. Significance is indicated above the whiskers as follows: *P < 0.05; **P < 0.01; ***P < 0.001. For the GWAS screens, mutations positively associated with MDR are aligned under the “+” sign whereas those either not associated with MDR (GWAS-1) or positively associated with antibiotic susceptibility (GWAS-2) are aligned under the “−” sign. The significance levels of the association with MDR (GWAS-1, + and −; GWAS-2, +) or susceptibility (GWAS-2, −) prior to and after correction for population structure are indicated within parentheses (uncorrected/corrected) below the mutations on the *x*-axis as follows: *P = 10^−06^–10^−10^; **P = 10^−10^–10^−20^; ***P = 10^−20^–10^−40^. See Table 2 for the complete GWAS-1 and GWAS-2 data. *NA* on the *x*-axis indicates that no mutation was found for the screen in the gene.

Fourteen genes had mutations in at least 2 of the 19 independent transformants (Table 1) and another 14 genes were mutated in only 1 of these 19 strains (Supplementary Table S4). We selected six shared genes based on functional diversity; *scrR* (SPV_1535) coding for a sucrose operon repressor, *purD* (SPV_0058) coding for a phosphoribosylamine-glycine ligase, *plsX* (SPV_0043) coding for a phosphate acyl-ACP acyltransferase, *pulA* (SPV_1002) coding for a type I pullulanase, *cps2O* (SPV_0331) coding for a dTDP-4-dehydrorhamnose reductase and SPV_1333 coding for a putative 6-phosphogluconolactonase, to study the role of their mutations in natural transformation. PCR fragments covering the mutations *scrR*^AG628A^ or coding for the PurD^E193K^, PlsX^H57Y^, PulA^W191*^, Cps2O^G70E^ or SPV_1333^P328S^ variants were amplified from the appropriate mutants and independently integrated in *S. pneumoniae* D39 by co-transformation with a 500 bp PCR fragment coding for RpsL^K56T^. STR-resistant colonies were reputed to have also integrated the mutation in the genes of interest and this was confirmed by Sanger sequencing. The *S. pneumoniae* D39-RpsL^K56T^, EscA^D232N^ and FruA^C508Y^ variants were also produced as potential negative controls of natural transformation. The gene *escA* codes for an ABC transporter that was mutated solely in T25 (Supplementary Table S4) and as such less likely to be phenotypic in contrast to genes mutated in independent transformants. Mutations in *fruA* were found in 3 of the 20 TMP-resistant mutants with increased transformation (Supplementary Figure S3A) but negative for FolA^I100L^ (M7, M10 and M13; Supplementary Table S4), and in one mutant incapable of natural transformation (N40; Supplementary Table S4). The D39 recombinants were grown as biofilms in C + Y and their capacities to naturally transform the FolA^I100L^ allele were tested. The *S. pneumoniae* D39-RpsL^K56T^, EscA^D232N^ and FruA^C508Y^ recombinants failed in acquiring FolA^I100L^, but *S. pneumoniae* with mutations in any of the six gene candidates could naturally transform to the FolA^I100L^ genotype with an efficiency ranging from 1 × 10^−2^ to 1 × 10^−5^ (Figure 2C). None of these mutations produced additional TMP resistance, the MIC of all recombinants being equal to D39 WT (2 μg/ml). The *S. pneumoniae* D39-PurD^E193K^ recombinant also transformed when assessed as planktonic cells (Figure 3A), although at a lesser efficiency than in biofilms (Figure 2C). Since working with planktonic cells was experimentally more amenable for mid-throughput analysis our additional work on natural transformation used planktonic cells.

**Table 1.** Mut-Seq derived mutations in genes detected in at least two *S. pneumoniae* D39 transformants

	Genes													
Mutants^a^	SPV_0043 *plsX*	SPV_0058 *purD*	SPV_0080 *pfbB*	SPV_0187 *nrdD*	SPV_0319 *cps2E*	SPV_0331 *cps2O*	SPV_0579 *cbpL*	SPV_0788 *dnaE*	SPV_1002 *pulA*	SPV_1330 *glnP*	SPV_1333	SPV_1437 *plsC*	SPV_1535 *scrR*	SPV_1787 *rnmV*
T3^b^				E462K						A161V				L164I
T14^c^	H57Y	E193K	E1062K						W191*		P328S	D242N		
T16^c^	H57Y	E193K	E1062K		Q213*				W191*		P328S	D242N		
T18^c^				E462K						A161V				L164I
T22^c^	H57Y	E193K	E1062K		Q213*				W191*		P328S	D242N	Indel^AG628A^	
T24^d^							G150S		W191*					
T25^d^							G150S							
T26^d^		E193K					G150S							
T28^e^						G70E		G879E						
T29^e^						G70E		G879E						
T32^e^	H57Y	E193K	E1062K						W191*		P328S	D242N		
T33^e^	H57Y	E193K	E1062K		Q213*				W191*		P328S	D242N	Indel^AG628A^	
T34^e^	H57Y	E193K	E1062K						W191*		P328S	D242N		
T35^e^	H7Y	E193K	E1062K		Q213*				W191*		P328S	D242N	Indel^AG628A^	

^a^Only mutations in genes detected in at least two *S. pneumoniae* D39 mutants are shown. Mutants had to have acquired the FolA^I100L^ genotype and to have increased natural transformation efficiency in C + Y. Five transformants (T15, T27, T30, T31 and T38) did not have recurrent mutations with other transformants.

^b–e^Mut-Seq screens were performed in four biological replicates. The transformants reported in this table were obtained during the first (^b^), second (^c^), third (^d^) and fourth (^e^) screens performed in C + Y with biofilms.

### Genes identified by Mut-Seq are also mutated in MDR *S. pneumoniae* clinical isolates

**Figure 4.**
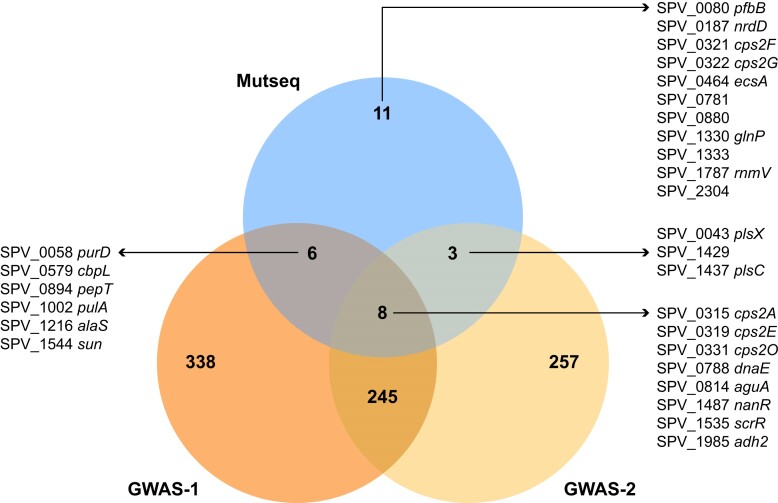
Intersection of genes revealed in the Mut-Seq screen linked to natural transformation and of GWAS-derived SNPs associated with MDR. A total of 28 different genes associated with increased natural transformation were highlighted by Mut-Seq (Supplementary Table S4), 17 of which were shared by at least one GWAS and associated with MDR. All SNPs associated with MDR in the GWAS can be found in Supplementary Table S6A and C.

We hypothesized that some of the genes identified using our Mut-Seq screen may also be mutated in MDR *S. pneumoniae* clinical isolates, as these are presumably highly transformable to serially acquire resistance genes. To test this, we performed a pilot GWAS on a small number of genomic sequences to detect nucleotide variants associated with MDR. The genomes of 108 MDR strains (resistant to three or more antibiotics) derived from our studies with Canadian (8) and Chinese (24) isolates along with sequences derived from a US study (32) were compared with those of 108 sensitive isolates derived mostly from the same US study (Supplementary Table S1B). This revealed 1538 mutations strongly associated with MDR (False Discovery Rate (FDR)-corrected *P*-value = 10^−46^–10^−7^), 65 of which being significant following adjustment for lineage effect (Supplementary Table S6A). The first genes ranking out from this GWAS were the core genes directly contributing to AMR such as *folA, pbp2x*, *pbp2b*, *pbp1a*, *sulA* and *sulB* (Supplementary Table S6A). Some genes not known to contribute directly to AMR were also highlighted by this GWAS and we focused on the intersection between Mut-Seq and this pilot GWAS (hereafter referred to as GWAS-1) (Figure 4) while applying selection criteria (see “Materials and methods” section). One shared gene was *purD* (Table 2; Supplementary Table S6A), for which the PurD^E193K^ variant derived from Mut-Seq was already validated to increase natural DNA transformation in *S. pneumoniae* D39 (Figure 2C). We introduced in *S. pneumoniae* D39 a PCR product coding for the PurD^S287A^ mutation revealed by GWAS-1. Assessing natural transformation among transformants grown as planktonic cells revealed that both GWAS-1 PurD^S287A^ and Mut-Seq PurD^E193K^ variants increased transformation efficiency in C + Y (Figure 3A). Neither D39 WT cells nor the D39-RpsL^K56T^ control transformed in C + Y medium (Figure 3). Another mutation, PurD^A157S^, was not associated with MDR isolates in GWAS-1 (Supplementary Table S6B). We hypothesized that its introduction in D39 would not lead to an increased rate of natural transformation, and this was indeed found to be the case (Figure 3A).

**Table 2.** Mutations in genes common to the Mut-Seq and to at least one GWAS of MDR isolates

Genes	Product	Mut-Seq mutations^a,b^	GWAS-1 mutations^a^	MDR/S^c^	GWAS-2 mutations^a^	MDR/S^c^
SPV_0043	PlsX	H57Y			A210G Q45P	47/2 (**/***) 47/3 (**/***)
SPV_0058	PurD	E193K	A120I S287A	61/4 (**/ns) 85/11 (**/*)		
SPV_0315	Cps2A	E193K	I409L L415I V477M	41/0 (**/ns) 51/7 (**/ns) 47/0 (**/ns)		
SPV_0319	Cps2E	Q213*	R239H EH387VQ P391S A242V T252N	44/0 (**/ns) 50/3 (**/ns) 50/3 (**/ns) 46/2 (**/ns) 50/5 (**/ns)	R234H R352C D343E G229D I219V L115R S231I T22A T5L V48A	46/6 (*/*) 66/13 (**/*) 47/6 (*/*) 47/6 (*/*) 47/6 (*/*) 47/6 (*/*) 47/6 (*/*) 47/6 (*/*) 47/6 (*/*) 46/6 (*/*)
SPV_0331	Cps2O	G70E	T37I M195I	47/0 (**/ns) 47/0 (**/ns)	K87R	42/3 (*/**)
SPV_0579	CbpL	G150S	ATT91STA A102S I84_N87delinsTTQTD S120N S295T S81_T82delinsA S89T	47/0 (**/ns) 67/9 (**/ns) 44/0 (**/ns) 67/10 (**/ns) 62/7 (**/ns) 44/0 (**/ns) 45/0 (**/ns)		
SPV_0788	DnaE	G879E	A649V	60/2 (**/ns)	E101V L153F T67I	35/0 (*/**) 41/2 (*/**) 35/0 (*/**)
SPV_0814	AguA	A298V	A138V P223S R141K S67Y	60/0 (***/ns) 60/0 (***/ns) 60/7 (**/ns) 60/0 (***/ns)	A269V L325I	48/5 (*/**) 93/5 (***/**)
SPV_0894	PepT	G108E	R9H T129M	59/3 (**/ns) 54/0 (**/ns)		
SPV_1002	PulA	W191*	E723D L714F	59/0 (**/ns) 59/0 (**/ns)		
SPV_1216	AlaS	E154K	A490T I241T S498P	69/6 (**/ns) 59/0 (**/ns) 70/10 (**/ns)		
SPV_1429		D73Y			G88H	45/6 (*/**)
SPV_1437	PlsC	D242N			R108C	43/5 (*/**)
SPV_1487	NanR	L245S	I9V T205S I112V T214A I127V S183T Q76E S257N T35Q	83/0 (***/ns) 82/0 (***/ns) 78/0 (***/ns) 78/0 (***/ns) 61/0 (**/ns) 60/0 (***/ns) 45/0 (**/ns) 37/0 (**/ns) 56/0 (**/ns)	Q76E I112V I127V I9V T205S T214A T35Q	87/1 (***/***) 80/0 (***/***) 76/0 (***/***) 105/1 (***/**) 111/1 (***/***) 74/1 (**/**) 53/0 (**/**)
SPV_1535	ScrR	Indel^AG628A^	V244I	60/0 (***/ns)	A3T	47/5 (*/**)
SPV_1544	SunL	R335Q	A135S	61/2 (**/ns)		
SPV_1985	Adh2	T186A	ANGH230ENGY	60/6 (**/ns)	S64R	44/5 (*/**)

^a^Mutations are at the protein level, except for Indels (small insertions/deletions) which are at the nucleotide level. The numbers indicate the position of the mutated amino acids in the protein. For Indels, numbers refer to the nucleotide position in the gene.

^b^Asterisks denote nonsense mutations.

^c^The number of genomes from MDR/susceptible (S) clinical strains harboring the mutation is indicated. Within parentheses are the Bonferroni-corrected significance of the association between the mutation and the MDR phenotype. *P = 10^−6^–10^−10^; **P = 10^−10^–10^−20^; ***P = 10^−20^–10^−40^ using Pyseer’s filter *P*-value unadjusted for population structure (left) and Pyseer’s lrt *P*-values that were adjusted for population structure (right). ns, not significant.

**Figure 5.**
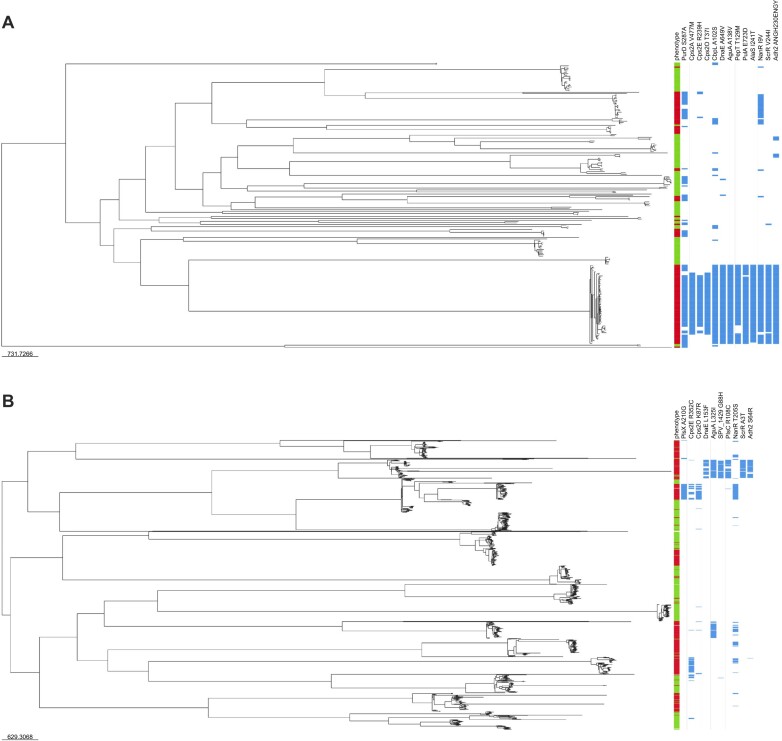
Phylogenetic distribution among MDR strains of GWAS mutations in genes also detected by Mut-Seq. Phylogeny was inferred from a recombination-pruned core SNP alignment of the 216 *S. pneumoniae* genomes used for our pilot GWAS-1 (**A**) and 850 *S. pneumoniae* genomes used for our GWAS-2 (**B**). Coloring at the tip of the branches refers to the antibiotic susceptibility status of the strains (red, AMR; green, susceptible). The heatmap on the right indicates the presence (blue) or absence (white) in the genomes of the mutations leading to the amino acid substitutions listed on top. Interactive versions of the figures are available at the following links: https://microreact.org/project/1uP9V6z4HzTheCNWDrM93M-spnmdr216august2024; https://microreact.org/project/oFajQo1ffGbFEVysRU1t3b-spnmdr850august2024.

Besides *purD*, we found 13 other genes common between Mut-Seq and GWAS-1 (Figure 4), although the exact mutations differed (Table 2). Several of these variants were clustered in the phylogenomic tree constructed from the 216 clinical isolates however, which affected the *P*-value after adjusting for lineage effect (Supplementary Table S6A), although some were spread in different MDR groups (Figure 5A). Moreover, mutations in 3 of these 13 genes pinpointed by Mut-Seq (ScrR^AG628A^, Csp2O^G70E^ and PulA^W191*^) were already proven to increase the natural DNA transformation of *S. pneumoniae* D39 in C + Y medium (Figure 2C). The GWAS-1 derived mutations Csp2O^T37I^ and Csp2O^D230G^, respectively associated or not with MDR (Table 2; Supplementary Table S6A and B), were introduced in *S. pneumoniae* D39 and incubated with a cell lysate containing FolA^I100L^. *S. pneumoniae* D39-Csp2O^T37I^, but not *S. pneumoniae* D39-Csp2O^D230G^, led to TMP-resistant colonies, hence exhibiting increased natural transformation (Figure 3C). This correlation between GWAS mutations in MDR isolates and natural transformation prompted us to assess the role of additional mutations despite the lineage effect in GWAS-1. We tested the sucrose operon repressor ScrR, for which the Mut-Seq derived ScrR^AG628A^ increased natural transformation in C + Y (Figure 2C). The GWAS-1 derived ScrR^V244I^ and ScrR^G190E^ mutations were associated and not associated with MDR, respectively (Supplementary Table S6A and B). The introduction in *S. pneumoniae* D39 of the GWAS-1 mutation ScrR^V244I^ (positively correlated to MDR), or of the Mut-Seq mutation ScrR^AG628A^, increased transformation efficiency in C + Y (Figure 3D). Significantly, the introduction of the ScrR^G190E^ mutation (not associated with MDR in GWAS-1) did not impact the rate of natural transformation (Figure 3D). We also introduced the GWAS-1 derived ScrR^V244I^ in CCRI-14647 and upon incubation with a *S. pneumoniae* D39-FolA^I100L^ lysate we observed that this strain, in contrast to its parent (Figure 1B), could transform in the C + Y medium (Supplementary Figure S4).

Many genes highlighted by GWAS-1 were not highlighted in our Mut-Seq screens (Figure 4). Among these, we noted mutations in seven genes of the *pur* operon that were significantly associated (Bonferroni-corrected *P*-value < 10^−17^–10^−32^) with MDR isolates (Supplementary Figure S5) in GWAS-1. The PurH^S158A^ variant had even a stronger association with MDR than the PurD^S287A^ mutation (Supplementary Figure S5). While *purH* was not a gene pinpointed by Mut-Seq, because of its strong association with MDR, and since it belongs to same pathway as PurD, we inserted the mutation coding for PurH^S158A^ in the genome of *S. pneumoniae* D39. We also introduced the PurH^V52M^ mutation, which was not associated with MDR (Supplementary Table S6B). These cells, when incubated with a lysate derived from *S. pneumoniae* D39-FolA^I100L^ indicated that the PurH^S158A^ transformant, but not the PurH^V52M^ transformant, exhibited increased natural transformation in C + Y (Figure 3B).

Overall, several mutations in genes highlighted by Mut-Seq and shared in GWAS-1, derived from a limited number of MDR strains and despite a phylogenetic bias, were shown experimentally to increase natural DNA transformation (see Figure 3). We increased the robustness of our approach by selecting 850 strains (half sensitive, half MDR) distinct from GWAS-1 to perform a second GWAS (GWAS-2). These strains were phylogenetically diverse (Figure 5B), belonging to 14 GPSCs (see “Materials and methods” section). After utilizing multidimensional scaling of genetic distances inferred from the phylogenetic tree to correct for population structure, the GWAS-2 revealed 513 mutations strongly associated with MDR (Supplementary Table S6C). Similarly to GWAS-1, the core genes directly contributing to resistance were associated significantly to MDR (Supplementary Table S6C). The two GWAS screens shared 253 genes that were mutated and associated with MDR (Figure 4). Collectively 17 genes (out of the 28 highlighted by Mut-Seq) were shared between the Mut-Seq screen and at least one of the GWAS (Figure 4). Of the six mutated genes derived from Mut-Seq validated to increase transformation (Figure 2C), *csp2O*, *scrR* and genes of the *pur* operon (either *purD* or *purH*) were highlighted by both GWAS, *pulA* and SPV_1333 were detected in GWAS-1 while *plsX* was part of GWAS-2 (Figure 4).

As we did for Mut-Seq and GWAS-1, we tested the role of GWAS-2 mutations for their capacity to increase natural transformation of *S. pneumoniae* D39 grown in C + Y, by focusing on genes or pathways common to GWAS-1 that we had investigated. Similarly, to GWAS-1 we noted mutations in four genes of the *pur* operon in GWAS-2 that were significantly associated (Bonferonni-corrected *P*-value < 10^−6^) with MDR isolates (Supplementary Figure S5). We focused on SNPs in *purD* and *purH*. The introduction of the mutation PurH^K451N^ (associated with MDR; Supplementary Table S6C) into the genome of *S. pneumoniae* D39 increased natural transformation (Figure 3B) while introducing PurD^S53Y^ or PurH^S494F^ (with lrt *P*-values associated with sensitive isolates; Supplementary Table S6D), did not. We expanded our work with the capsule gene *cps2O* that was also highlighted by Mut-Seq and the two GWAS. Introduction of the Cps2O^K87R^ mutation with lrt *P*-value associated with MDR (Table 2; Supplementary Table S6C) increased natural transformation of *S. pneumoniae* D39 in C + Y medium (Figure 3C), while introduction of Cps2O^K83E^ (with a lrt *P*-value of 9 × 10^−7^ associated with sensitive isolates; Supplementary Table S6D) did not impact natural transformation (Figure 3C). Similarly, the ScrR^A3T^ mutation associated with MDR isolates in GWAS-2 (lrt *P*-value 4 × 10^−16^; Table 2; Supplementary Table S6C), once introduced in *S. pneumoniae* D39, led to increased natural transformation (Figure 3D), while ScrR^E303K^ (with a lrt *P*-value of 1.7 × 10^−6^ associated with susceptibility; Supplementary Table S6D), did not impact natural transformation in the C + Y medium (Figure 3D).

## Discussion

Using a DNA lysate template for DNA transformation, a proxy of extracellular DNA present in the nasopharyngeal biofilm (33–35), we observed disparity in the ability of strains for natural transformation and this was conditioned by the growth medium (Figure 1). We exploited the inability of *S. pneumoniae* D39 for natural transformation in C + Y medium for a chemical mutagenesis screen (Figure 2). The mutagen EMS induces transitions (19) which limits the type of mutations that can be observed. However, it had a marked advantage in our screen since the generation of FolA^I100L^ and RpsL^K56T^ requires transversions (respectively, *folA* T298G and *rpsL* A167C). Thus, a FolA^I100L^ or RpsL^K56T^ genotype is most likely due to the result of transformation rather than mutagenesis. These mutagenized cells with increased natural transformation were characterized by genome sequencing, an approach that we used for studies of AMR (19,36,37). Mutations were pinpointed in a wide variety of genes and when selected mutations were introduced in the D39 background, an increase in natural transformation was observed (Figure 2C). Of great interest, our data support that adaptation for natural transformation also occurs in nature as determined by two GWAS screens. Indeed, MDR isolates, that are likely to have undergone a series of transformation cycles to accumulate drug resistance genes, have accumulated mutations in often the same genes as those revealed by Mut-Seq (Figure 4).

*S. pneumoniae* has close to 100 different polysaccharide capsule types encoded by the *cps* gene cluster with many genes arranged in tandem [reviewed in Paton and Trappetti (38)]. Several studies indicated that the capsule of *S. pneumoniae* diminishes DNA transformation (39,40). For example, a R379G mutation in Cps2E resulted in capsule loss and increased transformation Schaffner *et al.* (41). Interestingly, *cps2E* was mutated in our Mut-Seq screen and in GWAS (Figure 4 and Table 2). Capsule genes common to Mut-Seq and GWAS also include *cps2A* and *cps2O* (Figure 4 and Table 2) and introduction of the Cps2O^G70E^ mutation in D39 increased its natural transformation (Figure 2C). Mutations in *cps2F* and *cps2G* were found in many transformants derived from our Mut-Seq in C+Y medium (Supplementary Table S4). In our Mut-Seq screen, several clones did not have the expected FolA^I100L^ mutation but yet exhibited increased natural transformation (Supplementary Figure S3A). This suggests that selection for *S. pneumoniae* isolates with increased natural transformation is relatively straightforward (albeit here it was helped using EMS). Some of these clones had mutations in *cps2E*, *cps2F* and *cps2L* (Supplementary Table S4) possibly contributing to the increased natural transformation observed. The GWAS-2 revealed that multiple mutations in nine different *cps* genes were significantly associated with MDR (lrt *P*-value < 10^−7^; Supplementary Table S6C), suggesting that interfering with capsule production by various means increases natural transformation. Of note, a mutation in *cpsR* (SPV_0064; *cpsR*^V68I^), one of the two transcription factors repressing capsule biosynthesis in the airways (42), was highly significant (lrt *P*-value 2.4 × 10^−19^) in GWAS-2 (Supplementary Table S6B). It has been determined that the quantity of capsular polysaccharides modulate the rates of transformation and that nonencapsulated strains of *S. pneumoniae* transformed better than encapsulated ones (43). Mutations highlighted by Mut-Seq or in GWAS may affect capsule biosynthesis and impact the level of capsular polysaccharides and hence influence DNA transformation efficiency.

Mut-Seq revealed two genes implicated in phospholipid metabolism, *plsX* and *plsC* (Table 1). The introduction of PlsX^H57Y^ in D39 increased natural transformation in C + Y (Figure 2C). Both *plsX* and *plsC* (with respective lrt *P*-value of 8.6 × 10^−22^ and 2.3 × 10–^12^) were highlighted by GWAS-2 as associated with MDR (Supplementary Table S6B). Phospholipid metabolism is linked to DNA transformation in several bacteria including in *Bacillus subtilis* (44). In addition to capsular polysaccharides, phospholipids are major component of bacterial cell envelope. Mutations in *plsX* may alter phospholipids and impact membrane homeostasis and impact indirectly DNA transformation efficacy. Interestingly, a link between the competence induced cell–cell communication peptide BriC and phospholipid composition has been described (45). We also introduced the mutation SPV_1333^P328S^ in D39 which also led to increased natural transformation (Figure 2C). The annotation of SPV_1333 is unclear (46) and this gene was not highlighted in our GWAS (Supplementary Table S6). It is possible that requirements for natural transformation *in vitro* and *in vivo* may differ. Nonetheless, the variety and relative ease of selecting for mutations modulating natural transformation is consistent with the view that transformation is a way of life for *S. pneumoniae* as it allows the bacteria to adapt to different environments (47,48).

Genes of the purine operon including *purD* and *purH* were highlighted both by Mut-Seq (Table 1) and GWAS (Supplementary Figure S5). The Mut-Seq screen did not reveal a mutation in *purH* while GWAS-2 failed to find SNPs in *purD* significantly associated with MDR (Figure 4). The selective force between *in vitro* or *in vivo* conditions may differ. Yet, for all GWAS variants that were tested, those associated with MDR (PurD^S287A^, PurH^S158A^ and PurH^K451N^) increased natural transformation of *S. pneumoniae* D39 in C + Y, while those associated with susceptibility (PurD^A157S^, PurD^S53Y^, PurH^V52M^ and PurH^S494F^) did not (Figure 3). While the role of purines in *S. pneumoniae* transformation is unknown, the availability of purine nucleotides is known to regulate natural competence in *Haemophilus influenzae* by controlling the translation of a competence activator (49). Another gene pinpointed by Mut-Seq and by GWAS is *scrR*, coding for a sucrose operon repressor that regulates the expression of a phosphotransferase system (PTS) transporter operon (50). There is no published evidence linking *scrR* with DNA transformation. Similarly to the *pur* operon genes, all SNPs tested found in *scrR* and associated with MDR can lead to increased natural transformation in C + Y medium, while SNPs in *scrR* associated with susceptible isolates cannot (Figure 3D). Many polymorphisms in the same gene have the capacity to impact natural transformation.

The mechanistic forces behind the increased natural transformation are still to be elucidated. This may have to do with mutations having small fitness costs (or gains) that could produce signals leading to natural transformation. One obvious candidate would be modulation in expression of genes related to competence. However, the expression of the transcription factor of late phase competence *comX* (51) was in fact decreased in *S. pneumoniae* D39 with either PurD^E193K^ or PurD^S287A^ in comparison to control (Supplementary Figure S6). Further analysis will be required to understand how natural transformation is increased. Apparently, more than one polymorphism in the same gene can impact natural transformation and this may explain why we did not find common mutations between Mut-Seq and GWAS. The latter is further amplified by the use of EMS that leads to transitions while the GWAS mutations are both due to transitions and transversions. While many strains of *S. pneumoniae* are reputed to be non-naturally transformable (52–54) our this work suggests that the use of different media (Figure 1) may indeed lead to natural transformation. Follow-up studies will be important to deepen the mechanistic underlying natural transformation. With many genes involved, it is more likely to come from generic and subtle changes (e.g. fitness, stress and alteration in metabolism).

In summary, this study demonstrated that natural transformation in *S. pneumoniae* is achievable under laboratory conditions, even with planktonic cells, pending that the right combination of genotype and medium are used. Mutations in a wide variety of gene products can increase the ability of a strain for natural transformation in a new environment. This also appears to take place in nature, where SNPs in noncore resistance gene associated, through GWAS analysis, with MDR strains were also shown to increase natural DNA transformation. While some of the mutations may be involved in resistance (55), experimental work presented here suggests that some may in all likelihood have been selected for facilitating the acquisition of DNA including resistance genes/mutations.

## Supplementary Material

gkae1140_Supplemental_Files

## Data Availability

The sequencing reads for the genomes sequenced as part of this study have been deposited to the Sequence Read Archive under BioProject accession PRJNA1021501 and biosample accessions are included in Supplementary Table S1A.
