# Supplementary material for: Point mutations in functionally diverse genes are associated with increased natural DNA transformation in multidrug resistant Streptococcus pneumoniae
Source: Nucleic Acids Res. 2024 Dec 3;53(1):gkae1140. doi: 10.1093/nar/gkae1140 (PMC11724299; doi:10.1093/nar/gkae1140)
Supplement: gkae1140_Supplemental_Files [file gkae1140_supplemental_files.zip › SupplementaryFigures.pdf]

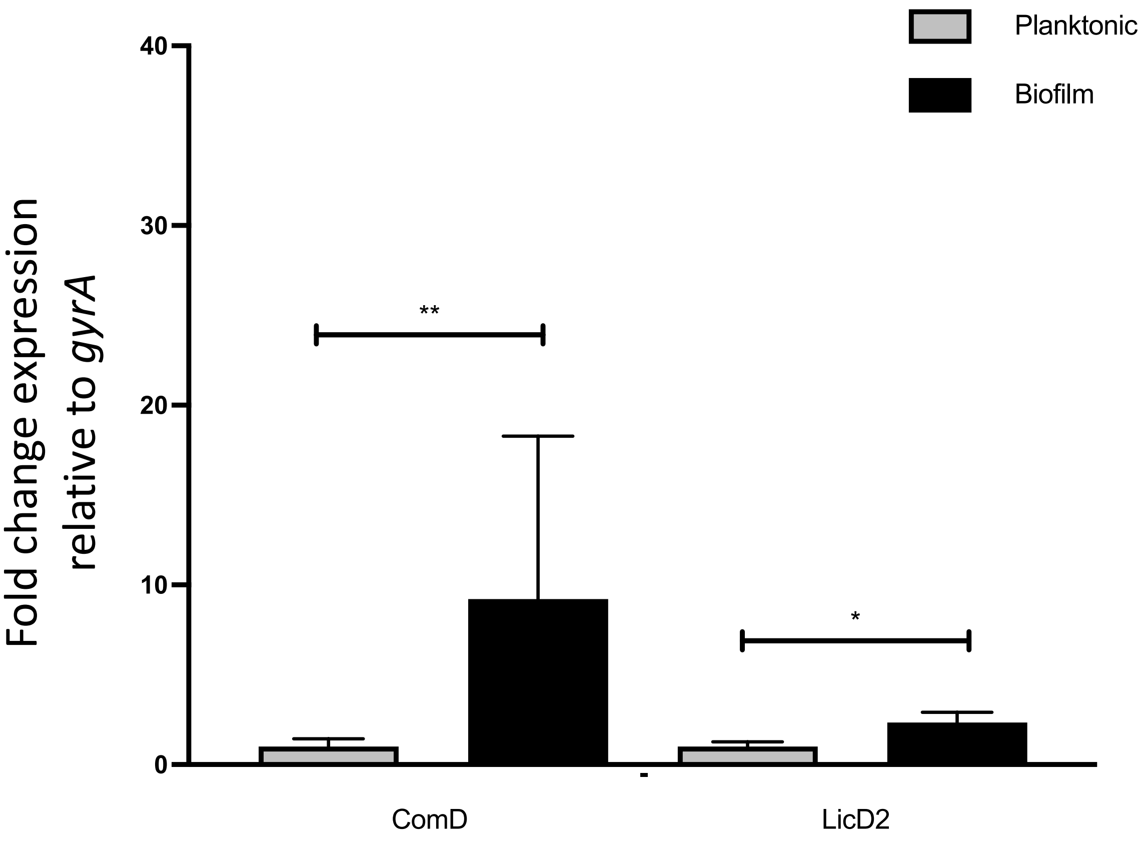

Figure S1. The *comD* and *licD2* genes are overexpressed in biofilm compared to D39 planktonic cells. Quantitative RT-PCR showing relative *S. pneumoniae* D39 *comD* or *licD2* expression compared with cells grown as planktonic cells or as biofilm. All qRT-PCR data were normalized according to the amplification signals of the housekeeping *gyrA* mRNA. Errors bars indicate the standard deviation for at least triplicate measurements. Expressions were compared using the Mann-Whitney test. *, $p < 0.05$; **, $p<0.01$.

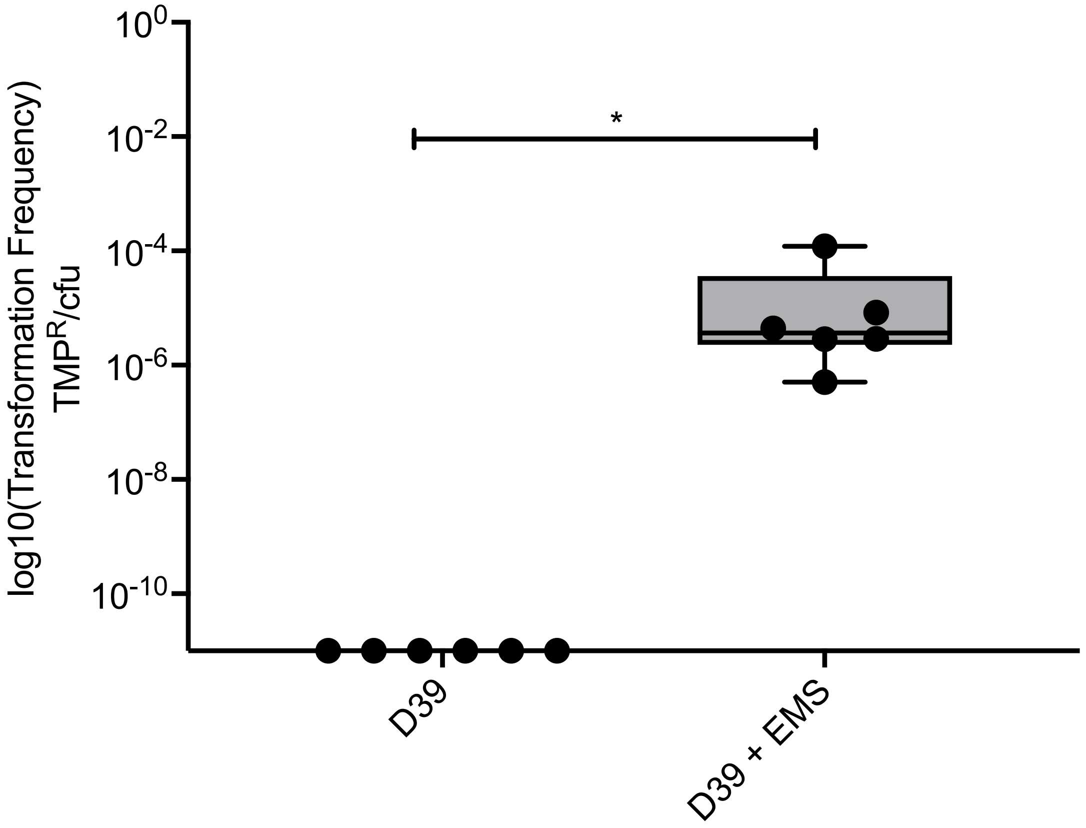

Figure S2. **Mutagenesis allows to select for *S. pneumoniae* D39 biofilms with increased natural transformation capacity in C+Y medium**. *S. pneumoniae* D39 was mutagenized by exposure to EMS and its natural transformation efficiency when grown as biofilm in C+Y compared to non-mutagenized control cells. Transformation efficiency was tested following exposure to a D39-FolA^{I100L} lysate, which confers resistance to trimethoprim (TMP), and is reported as the number of TMP-resistant cells over the number of viable cells. Each point represents a biological replicate. Transformation efficiencies were compared using the Mann-Whitney test. *, $p < 0.05$.

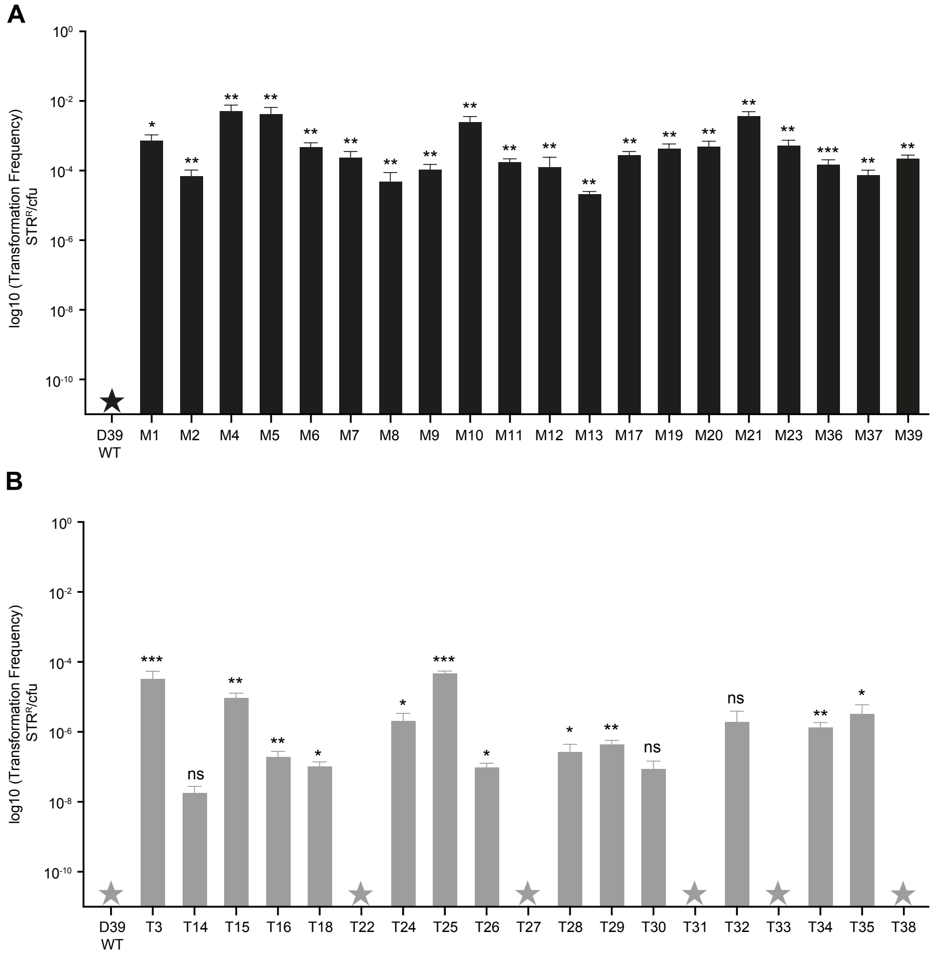

Figure S3. Natural transformation of mutagenized *Streptococcus pneumoniae* D39 strain grown as biofilms and as planktonic cells in C+Y.

The natural transformation efficiency of the 20 *S. pneumoniae* D39 mutants without the $FolA^{I100L}$ genotype derived from our Mut-Seq screen was assessed as biofilm in C+Y following exposure to a D39-$RpsL^{K56T}$ lysate (A). The natural transformation efficiency of 19 *S. pneumoniae* D39 hyper-transformants with the $FolA^{I100L}$ genotype derived from our Mut-Seq screen was cultivated as planktonic in C+Y following exposure to a D39-$RpsL^{K56T}$ lysate (B). Transformation efficiency is reported as the number of STR-resistant transformants over the number of viable cells. Error bars represent the standard error calculated for biological replicates (n=6). Transformation efficiencies were compared to the one of D39 WT using the Kruskal-Wallis test followed by Dunn's test. *, $p < 0.05$; **, $p < 0.01$; ***, $p < 0.001$, ns: not significant.

★ Transformation events not detected.

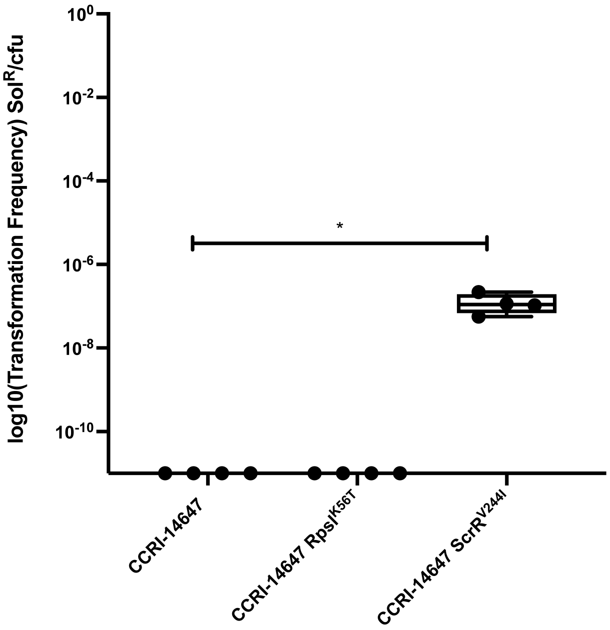

Figure S4. Mutation in ScrR from GWAS increase transformation in *S. pneumoniae CCRI-14647*. The mutation ScrR^{V244I} detected by GWAS was introduced in the genome of *S. pneumoniae* CCRI-14647. The natural transformation efficiency of the transformants was monitored with cells grown in C+Y media following exposure to a cell lysate derived from *S. pneumoniae* D39 coding for the S4^{C155T} variant producing resistance to solithromycin (Gingras et al., 2023). The use of this marker was necessary since CCRI-14647 is resistant to trimethoprim. Transformation efficiency is reported as the number of Solithromycin-resistant cells over the number of viable cells. Each point represents a biological replicate. Transformation efficiencies were compared to the one of CCRI-14647 using the Kruskal-Wallis test followed by Dunn's test. *, $p < 0.05$; **, $p < 0.01$; ***, $p < 0.001$; ****, $p < 0.0001$.

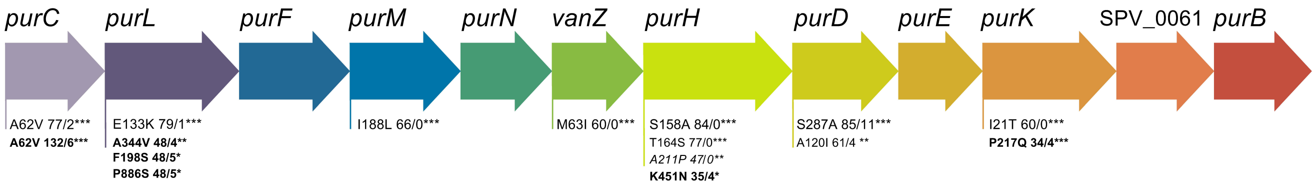

Figure S5. The *pur* operon of *Streptococcus pneumoniae* D39. The *pur* operon contains 11 genes whose names appear on top of the arrows. Seven of those had at least one mutation significantly associated with MDR isolates in our 216 GWAS-1 genomes and four of those had at least one mutation significantly associated with MDR isolates in our 850 genomes GWAS-2 genomes (mutations in bold) (*, p ranging from 10^{-06} to 10^{-10}; **, p ranging from 10^{-10} to 10^{-20}; ***, p ranging from 10^{-20}-10^{-40}). Below the genes is the position of amino acids changes. At the right of the mutation is the number of MDR isolates and the number of sensitive isolates in which the mutation was detected. For example, the PurH mutation S158A was found in 84 MDR isolates (out of 108) and in 0 sensitive ones (out of 108) in the GWAS-1.

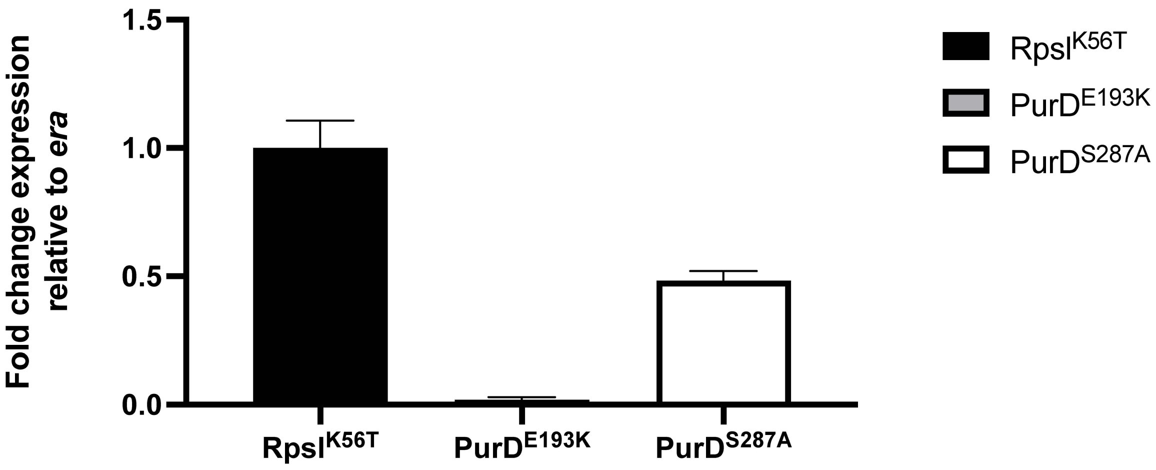

Figure S6. Comparative analysis of *comX* gene expression. Quantitative RT-PCR from RNA isolated at OD_{600} 0.1 showing the relative expression of *comX* in *S. pneumoniae* D39 *purD* mutants compared with D39-RpsL^{K56T}. All qRT-PCR data was normalized according to the amplification signals of the housekeeping *era* mRNA. Errors bars indicate the standard deviation for at least triplicate measurements.
