# Supplementary material for: Point mutations in functionally diverse genes are associated with increased natural DNA transformation in multidrug resistant Streptococcus pneumoniae
Source: Nucleic Acids Res. 2024 Dec 3;53(1):gkae1140. doi: 10.1093/nar/gkae1140 (PMC11724299; doi:10.1093/nar/gkae1140)
Supplement: gkae1140_Supplemental_Files [file gkae1140_supplemental_files.zip › Table S2.docx]

**Table S2. Primers used for introducing SNPs in *S. pneumoniae* D39 and CCRI-14647**

| Primers to introduce in D39 mutations detected in mutagenized mutants | | | |
| --- | --- | --- | --- |
| Protein^amino acid substitution^ | Mutant_Source of the mutated PCR fragment | Primers | 5'-3' |
| RpsL^K56T^ | CP1296 (Sung, et al. ^26^) | RPSL_5kb_Fw  RPSL_5kb_Rv  RPSL_500bp_Fw  RPSL_500bp_Rv | TAAATTCTGCGATTCGTTTT  GACCTTTACCACCAGACTGA  TGTACAGGGACGTGCTGACAA  GATAAGACGAGTAACTAGTTGTGAAT |
| FolA^I100L^ | R6-DHFR^I100L^ (Gingras, et al. ^24^) | DHFR_5kb_Fw  DHFR_5kb_Rv | GCCAACGATTTTTCCTTGAA  GGGAACCGAACATGCTAAGA |
| Cps2O^G70E^ | T28 | FPF182  FPF183 | GACGATAGCGTTGCTTTAA  GAAATCTTTGATTTCCCCTTTTAC |
| PurD^E193K^ | T14 | FPF180  FPF181 | TCTTGATTAGTTTATTGTTTGAGTG  ACCGAGGACATTAAGCATAA |
| PlsX^H57Y^ | T14 | FPF189  FPF190 | GTGGAAAGCTATTCTCTAATGTATT  TACTATGACTTAGCTAGACAACTTT |
| SPV_1333^P328S^ | T14 | FPF268  FPF269 | GATAGAAACCAATCCCTTCAAG  TTTGATGAGCTTTATGTTTGCT |
| ScrR^AG628A^ | T22 | FPF244  FPF245 | AAAGATAATTTGGTATTGCCCT  GTGTAGCTATGTTGATTACACAAA |
| PulA^W191*^ | T14 | FPF287  FPF288 | CAGGACAAATCGATTTCTAACA  TCGTTTGCAAGAAGAGGATT |
| FruA^C508Y^ | M10 | FPF232  FPF233 | TTCGAAAACCAGTCAGATCT  TTCGAAAACCAGTCAGATCT |
| EcsA^D232N^ | T25 | FPF251  FPF252 | ATTTTCTCAAGTTTCTTCGGCG  GATGTTCCTTCAAGAGTTTCTC |
| Primers to introduce in D39 mutations detected by GWAS | | | |
| Protein^amino acid substitution^ | Primers | 5'-3' | |
| PurD^S287A^ | FPF304  FPF300  FPF301  FPF305 | TGGATCAATATCTTAGGTATGCTTAGC  AGCAGACGGGCCGAAGGTCATCGAGTTCAACGCTCGATTT  AAATCGAGCGTTGAACTCGATGACCTTCGGCCCGTCTGCT  TTGTACCTTATCTTGGTCAGCAC | |
| PurD^A157S^ | FPF180  CG112  CG113  FPF181 | TCTTGATTAGTTTATTGTTTGAGTG  GAAGGGTGTCGTCGTT**T**CTGAGACGGTTGAGC  GCTCAACCGTCTCAG**A**AACGACGACACCCTTC  ACCGAGGACATTAAGCATAA | |
| PurD^S53Y^ | CG98  CG100  CG101  CG99 | GGTTGTATCCGGAAGTAGTGAAGGCTC  GTAAATATCTCTATTTCCGAACATT**A**TAAATTGATTGACTTCGCAAAGACC  GGTCTTTGCGAAGTCAATCAATTTA**T**AATGTTCGGAAATAGAGATATTTAC  GCATAGGCTGCTTCCAAGTCTGC | |
| PurH^S158A^ | FPF310  FPF308  FPF309  FPF311 | GCT TTG GTG GTA TGT TTG ACC TTT  GTT TTG GAC GAG TTG GCA GCCAATGGTGAAACGACTTACG  CGTAAGTCGTTTCACCATTGGCTGCCAACTCGTCCAAAAC  TTCACTGTTCTGGTCTTTTCACCA | |
| PurH^V52M^ | CG135  CG137  CG138  CG136 | GCTTGATTGAGTCGATTGG  GGAGACCATTGCGATCG**A**TGATATGACGGGCTTCC  GGAAGCCCGTCATATCA**T**CGATCGCAATGGTCTCC  CATGATATTTGGCTCCTTGC | |
| PurH^K451N^ | CG114  CG116  CG117  CG115 | GTGATTGCCGAAGAATTTCC  CATTGACCAAGCCAA**T**GATCGTCTGGACGGG  CCCGTCCAGACGATC**A**TTGGCTTGGTCAATG  CATCATCTGACCCAGTTGA | |
| PurH^S494F^ | CG139  CG140  CG141  CG115 | GTTGGACCAACCTTATTGTC  GCTCTGTCCGTGACCAAGAAT**T**CATCGAAGCAGC  GCTGCTTCGATG**A**ATTCTTGGTCACGGACAGAGC  CATCATCTGACCCAGTTGA | |
| Cps2O^T37I^ | CG129  CG130  CG131  CG80 | ATGGTTCCGAATTTGGGAT  GTGGCTGAGATGGACATTA**T**CGATGCAGAAATGGTT  AACCATTTCTGCATCG**A**TAATGTCCATCTCAGCCAC  TTTACATAGGCATCCAACCC | |
| Cps2O^D230G^ | CG125  CG132  CG133  CG134 | TGTTCAAGTTGCCAATTTGG  TTGCAGTTGAAATTTTGAAAG**G**TACAGATGTCGAAGTCAAGCC  GGCTTGACTTCGACATCTGTA**C**CTTTCAAAATTTCAACTGCAA  GTCAATATTTGTACCGACTAGATAC | |
| Cps2O^K87R^ | CG79  CG81  CG82  CG80 | GGTTACCAAGTGAAGGATCC  CGGGGACAAAAAATGTCGCAA**G**AGCATCTGAAAAGCAT  ATGCTTTTCAGATGCT**C**TTGCGACATTTTTTGTCCCCG  TTTACATAGGCATCCAACCC | |
| Cps2O^K83E^ | CG79  CG105  CG106  CG80 | GGTTACCAAGTGAAGGATCC  CATCAATGTGACGGGGACA**G**AAAATGTCGCAAAAGCATC  GATGCTTTTGCGACATTTT**C**TGTCCCCGTCACATTGATG  TTTACATAGGCATCCAACCC | |
| ScrR^V244I^ | FPF371  FPF373  FPF372  FPF374 | TTT GTT CTT GTT TGA AAG CG  TTCGGATGATTTGACAGCTATTCTGATCATTAAAATCGCT  AGCGATTTTAATGATCAGAATAGCTGTCAAATCATCCGAA  AGCAAGAAAGACAAGAAGTT | |
| ScrR^G190E^ | FPF244  CG121  CG122  FPF245 | AAAGATAATTTGGTATTGCCCT  CAATTCGCCAACCG**A**ACTGCGCCACGCTG  CAGCGTGGCGCAGT**T**CGGTTGGCGAATTG  GTGTAGCTATGTTGATTACACAAA | |
| ScrR^A3T^ | CG84  CG86  CG87  CG85 | ACAACAGGGATGAAACCAA  TTACTATGAATTAGATTATGGTCACAAAACTAACTGATGTCGCCAAA  TTTGGCGACATCAGTTAGTTTTGTGACCATAATCTAATTCATAGTAA  CCTATCCGTTGCAGACTATG | |
| ScrR^E303K^ | CG102  CG103 CG104  FPF374 | GGAATGAATGGAATGGACAAC  CAAAAGATTGAAGGCAAG**A**AAGTCGCCACAACTGGTT  AACCAGTTGTGGCGACTT**T**CTTGCCTTCAATCTTTTG  AGCAAGAAAGACAAGAAGTT | |
| Primers to introduce in CCRI-14647 mutations detected by GWAS | | | |
| Protein^amino acid substitution^ | Primers | 5'-3' | |
| ScrR^V244I^ | FPF371  FPF372  FPF373  FPF374 | TTTGTTCTTGTTTGAAAGCG  AGCGATTTTAATGA**T**CAGAATAGCTGTCAAATCATCCGAA  TTCGGATGATTTGACAGCTATTCTGATCATTAAAATCGCT  AGCAAGAAAGACAAGAAGTT | |
| Primers for RT-PCR | | | |
| Gene targeted | Primers | 5'-3' | |
| *comD* | comD_FW  comD_RV | GATGGGTTAGGGATTGAAAGAT  AGTTACGGACAGAAAGTTATGAG | |
| *licD2* | licD2_FW  licD2_RV | ACGAGCAGTTCACGGTGATAGCAA  ATCCCTTCCTTACCGATCCCAACT | |
| *gyrA* | gyrA_FW4  gyrA_RV4 | ATCAATCAGCCGTTCGTGGT  CAAGCTGGCACCAACAACTG | |
| *comX* | comX_FW2  comX_RV2 | TGGGAATTGTCGGATTGGGA  ACGCTTCTGACTTTCCTGCT | |
| *era* | era_Fw  era_Rv | GTAGTATGGCCCGTCGTGAT  TAGCTTTTTATCGCGCCAGT | |
