# Supplementary material for: Point mutations in functionally diverse genes are associated with increased natural DNA transformation in multidrug resistant Streptococcus pneumoniae
Source: Nucleic Acids Res. 2024 Dec 3;53(1):gkae1140. doi: 10.1093/nar/gkae1140 (PMC11724299; doi:10.1093/nar/gkae1140)
Supplement: gkae1140_Supplemental_Files [file gkae1140_supplemental_files.zip › Table S4.docx]

**Table S4. Mutations in coding regions among *S. pneumoniae* D39V clones after EMS chemical mutagenesis.**

| **Mutant** | **Mutations^a^** | **Gene name** |
| --- | --- | --- |
| **Hyper-transformants with increased natural transformation with the FolA^I100L^ genotype** | | |
| T3 | SPV_0187 **G1390A** E462K  SPV_1330 **G482A** A161V  SPV_1401 **T298G** I100L  SPV_1787 **G490T** L164I | Ribonucleotide reductase of class III (anaerobic), large subunit ***nrdD***  ABC transporter, permease protein ***glnP***  Dihydrofolate reductase ***folA***  Ribonuclease M5 ***rnmV*** |
| T14 | SPV_0043 **C169T** H57Y  SPV_0058 **G577A** E193K  SPV_0080 **G3184A** E1062K  SPV_1002 **C573T** W191*  SPV_1333 **C982T** P328S  SPV_1401 **T298G** I100L  SPV_1437 **G724A** D242N | Phosphate:acyl-ACP acyltransferase ***plsX***  Phosphoribosylamine--glycine ligase ***purD***  Cell wall surface anchor family protein ***pavB/pfbB***  Predicted glycogen debranching enzyme (pullulanase-like, but lacking signal peptide) ***pulA***  Hypothetical protein  Dihydrofolate reductase ***folA***  Acyl-ACP:1-acyl-sn-glycerol-3-phosphate acyltransferase ***plsC*** |
| T15 | SPV_1401 **T298G** I100L  SPV_1544 **C1004T** R334Q  SPV_1985 **T556C** T186A | Dihydrofolate reductase ***folA***  16S rRNA (cytosine(967)-C(5))-methyltransferase ***sunL***  Lactaldehyde dehydrogenase involved in fucose or rhamnose utilization ***adh2*** |
| T16 | SPV_0043 **C169T** H57Y  SPV_0058 **G577A** E193K  SPV_0080 **G3184A** E1062K  SPV_0319 **C637T** Q213*  SPV_1002 **C573T** W191*  SPV_1333 **C982T** P328S  SPV_1401 **T298G** I100L  SPV_1437 **G724A** D242N | Phosphate:acyl-ACP acyltransferase ***plsX***  Phosphoribosylamine--glycine ligase ***purD***  Cell wall surface anchor family protein ***pavB/pfbB***  Undecaprenyl-phosphate galactosephosphotransferase ***cps2E***  Predicted glycogen debranching enzyme (pullulanase-like, but lacking signal peptide) ***pulA***  Hypothetical protein  Dihydrofolate reductase ***folA***  Acyl-ACP:1-acyl-sn-glycerol-3-phosphate acyltransferase ***plsC*** |
| T18 | SPV_0187 **G1390A** E462K  SPV_0894 **G323A** G108E  SPV_1216 **C460T** E154K  SPV_1330 **G482A** A161V  SPV_1401 **T298G** I100L  SPV_1787 **G490T** L164I | Ribonucleotide reductase of class III (anaerobic), large subunit ***nrdD***  Tripeptide aminopeptidase ***pepT***  Alanyl-tRNA synthetase ***alaS***  ABC transporter, permease protein ***glnP***  Dihydrofolate reductase ***folA***  Ribonuclease M5 ***rnmV*** |
| T22 | SPV_0043 **C169T** H57Y  SPV_0058 **G577A** E193K  SPV_0080 **G3184A** E1062K  SPV_0319 **C637T** Q213*  SPV_1002 **C573T** W191*  SPV_1333 **C982T** P328S  SPV_1401 **T2998G** I100L  SPV_1437 **G724A** D242N  SPV_1535 **AG628A^b^** | Phosphate:acyl-ACP acyltransferase ***plsX***  Phosphoribosylamine--glycine ligase ***purD***  Cell wall surface anchor family protein ***pavB/pfbB***  Undecaprenyl-phosphate galactosephosphotransferase ***cps2E***  Predicted glycogen debranching enzyme (pullulanase-like, but lacking signal peptide) ***pulA***  Hypothetical protein  Dihydrofolate reductase ***folA***  Acyl-ACP:1-acyl-sn-glycerol-3-phosphate acyltransferase ***plsC***  Sucrose operon repressor, LacI family ***scrR*** |
| T24 | SPV_0579 **G448A** G150S  SPV_1022 **C573T** W191*  SPV_1401 **T298G** I100L | Endo-beta-N-acetylglucosaminidase ***cbpL***  Predicted glycogen debranching enzyme (pullulanase-like, but lacking signal peptide) ***pulA***  Dihydrofolate reductase ***folA*** |
| T25 | SPV_0464 **G694A** D232N  SPV_0579 **G448A** G150S  SPV_1401 **T298G** I100L | ABC transporter ATP-binding protein EcsA, SHP exporter ***ecsA***  Endo-beta-N-acetylglucosaminidase ***cbpL***  Dihydrofolate reductase ***folA*** |
| T26 | SPV_0058 **G577A** E193K  SPV_0579 **G448A** G150S  SPV_1401 **T298G** I100L | Phosphoribosylamine--glycine ligase ***purD***  Endo-beta-N-acetylglucosaminidase ***cbpL***  Dihydrofolate reductase ***folA*** |
| T27 | SPV_0315 **G577A** E193K  SPV_0322 **A805G** K269E  SPV_0814 **C893T** A298V  SPV_1401 **T298G** I100L  SPV_1429 **C217A** D73Y  SPV_1487 **T734C** L245S | Exopolysaccharide biosynthesis transcriptional regulator ***cps2A***  Glycosyltransferase ***cps2G***  Agmatine deiminase ***aguA***  Dihydrofolate reductase ***folA***  Hypothetical protein  Sialic acid utilization regulator, RpiR family ***nanR*** |
| T28 | SPV_0788 **G2663A** G879E  SPV_0331 **G209A** G70E  SPV_1401 **T298G** I100L | DNA polymerase III alpha subunit ***dnaE***  dTDP-4-dehydrorhamnose reductase ***cps2O***  Dihydrofolate reductase ***folA*** |
| T29 | SPV_0331 **G209A** G70E  SPV_0788 **G2663A** G879E  SPV_1401 **T298G** I100L | dTDP-4-dehydrorhamnose reductase ***cps2O***  DNA polymerase III alpha subunit ***dnaE***  Dihydrofolate reductase ***folA*** |
| T30 | SPV_1401 **T298G** I100L | Dihydrofolate reductase ***folA*** |
| T31 | SPV_1401 **T298G** I100L | Dihydrofolate reductase ***folA*** |
| T32 | SPV_0043 **C169T** H57Y  SPV_0058 **G577A** E193K  SPV_0080 **G3184A** E1062K  SPV_1002 **C573T** W191*  SPV_1333 **C982T** P328S  SPV_1401 **T298G** I100L  SPV_1437 **G724A** D242N | Phosphate:acyl-ACP acyltransferase ***plsX***  Phosphoribosylamine--glycine ligase ***purD***  Cell wall surface anchor family protein ***pavB/pfbB***  Predicted glycogen debranching enzyme (pullulanase-like, but lacking signal peptide) ***pulA***  Hypothetical protein  Dihydrofolate reductase ***folA***  Acyl-ACP:1-acyl-sn-glycerol-3-phosphate acyltransferase ***plsC*** |
| T33 | SPV_0043 **C169T** H57Y  SPV_0058 **G577A** E193K  SPV_0080 **G3184A** E1062K  SPV_0319 **C637T** Q213*  SPV_1002 **C573T** W191*  SPV_1333 **C982T** P328S  SPV_1401 **T298G** I100L  SPV_1437 **G724A** D242N  SPV_1535 **AG628A^b^** | Phosphate:acyl-ACP acyltransferase ***plsX***  Phosphoribosylamine--glycine ligase ***purD***  Cell wall surface anchor family protein ***pavB/pfbB***  Undecaprenyl-phosphate galactosephosphotransferase ***cps2E***  Predicted glycogen debranching enzyme (pullulanase-like, but lacking signal peptide) ***pulA***  Hypothetical protein  Dihydrofolate reductase ***folA***  Acyl-ACP:1-acyl-sn-glycerol-3-phosphate acyltransferase ***plsC***  Sucrose operon repressor, LacI family ***scrR*** |
| T34 | SPV_0043 **C169T** H57Y  SPV_0058 **G577A** E193K  SPV_0080 **G3184A** E1062K  SPV_1002 **C573T** W191*  SPV_1333 **C982T** P328S  SPV_1401 **T298G** I100L  SPV_1437 **G724A** D242N | Phosphate:acyl-ACP acyltransferase ***plsX***  Phosphoribosylamine--glycine ligase ***purD***  Cell wall surface anchor family protein ***pavB/pfbB***  Predicted glycogen debranching enzyme (pullulanase-like, but lacking signal peptide) ***pulA***  Hypothetical protein  Dihydrofolate reductase ***folA***  Acyl-ACP:1-acyl-sn-glycerol-3-phosphate acyltransferase ***plsC*** |
| T35 | SPV_0043 **C169T** H57Y  SPV_0058 **G577A** E193K  SPV_0080 **G3184A** E1062K  SPV_0319 **C637T** Q213*  SPV_1002 **C573T** W191*  SPV_1333 **C982T** P328S  SPV_1401 **T298G** I100L  SPV_1437 **G724A** D242N  SPV_1535 **AG628A^b^** | Phosphate:acyl-ACP acyltransferase ***plsX***  Phosphoribosylamine--glycine ligase ***purD***  Cell wall surface anchor family protein ***pavB/pfbB***  Undecaprenyl-phosphate galactosephosphotransferase ***cps2E***  Predicted glycogen debranching enzyme (pullulanase-like, but lacking signal peptide) ***pulA***  Hypothetical protein  Dihydrofolate reductase ***folA***  Acyl-ACP:1-acyl-sn-glycerol-3-phosphate acyltransferase ***plsC***  Sucrose operon repressor, LacI family ***scrR*** |
| T38 | SPV_0321 **C101T** S25F  SPV_0781 **C166T** L56F  SPV_0781 **C1061T** P354L  SPV_0880 **A1712G** E571G  SPV_1401 **T298G** I100L  SPV_2304 **G344A** A115V | Alpha-L-Rha alpha-1,3-L-rhamnosyltransferase ***cps2F***  Hypothetical protein  Hypothetical protein  Hypothetical protein  Dihydrofolate reductase ***folA***  ISSpn7 transposase |
| **Mutants with increased natural transformation but without the FolA^I100L^ genotype^c^** | | |
| M1 | SPV_1834 **C1040A** G347V  SPV_1401 **C93A** L31F | Bifunctional acetaldehyde dehydrogenase/alcohol dehydrogenase ***adh***  Dihydrofolate reductase ***FolA*** |
| M2 | SPV_0349 **C406T** P136S  SPV_0666 **G421A** D141N  SPV_1007 **C958T** D320N  SPV_1320 **C778T** D260N | Isopentenyl-diphosphate delta-isomerase, FMN-dependent ***fni***  DNA polymerase III delta subunit ***holA***  Glycogen biosynthesis glucose-1-phosphate adenylyltransferase subunit ***glgD***  Hypothetical protein |
| M4 | SPV_0319 **A572AG^b^** | Undecaprenyl-phosphate galactosephosphotransferase **c*ps2E*** |
| M5 | SPV_0925 **C701T** G234D  SPV_1100 **G1251T** L417F | Hypothetical protein  Glucose-6-phosphate 1-dehydrogenase ***zwf*** |
| M6 | SPV_1033 **C476A** S159Y  SPV_1230 **G644A** S215L  SPV_1395 **G172C** L58V  SPV_1668 **G155A** S52F | Hypothetical protein  Phosphate transport system permease protein ***ptsA***  Hypothetical protein  Peptide ABC transporter ATP-binding protein ***amiE*** |
| M7 | SPV_0367 **T24A** L8F  SPV_0773 **G2648A** A580T  SPV_0444 **G1738A** G883D  SPV_0939 **G118A** E40K  SPV_1115 **C478T** E160K  SPV_1999 **G614A** A205V | Signal peptidase I ***lepB***  Fructose-specific PTS IIABC components ***fruA***  Endo-beta-N-acetylglucosaminidase ***endoD***  MutR family transcriptional regulator ***rgg***  3-isopropylmalate dehydrogenase ***leuB***  Zinc ABC transporter, ATP-binding protein ***adcC*** |
| M10 | SPV_0773 **G1523A** C508Y | Fructose-specific PTS IIABC components ***fruA*** |
| M11 | SPV_0321 **C567A** N180K  SPV_0768 **C907A** P303T | Alpha-L-Rha alpha-1,3-L-rhamnosyltransferase ***cps2F***  Coordinator of zonal cell elongation ***cozE*** |
| M12 | SPV_1334 **A342ATGT^b^**  SPV_1679 **G37T** D13Y | ATP synthase F1 sector epsilon chain ***atpC***  Multiple sugar metabolism operon regulatory protein ***msmR/rafR*** |
| M13 | SPV_0773 **G1738A** A580T  SPV_1345 **G173A** A58V  SPV_1395 **G572T** P191Q  SPV_1667 **C832T** E278K  SPV_1679 **G67T** E23* | Fructose-specific PTS IIABC components ***fruA***  Transcription elongation factor ***greA***  Hypothetical protein  Peptide ABC transporter ATP-binding protein ***amiF***  Multiple sugar metabolism operon regulatory protein ***msmR/rafR*** |
| M17 | SPV_0058 **G577A** E193K  SPV_1002 **C573T** W191* | Phosphoribosylamine--glycine ligase ***purD***  Predicted glycogen debranching enzyme (pullulanase-like, but lacking signal peptide) ***pulA*** |
| M19 | SPV_0328 **A611G** Y204C  SPV_1100 **G1251T** L417F  SPV_1211 **C457T** D153N  SPV_1375 **G175A** A59T | Glucose-1-phosphate thymidylyltransferase ***cps2L***  Glucose-6-phosphate 1-dehydrogenase ***zwf***  3-dehydroquinate dehydratase I ***aroD***  Hypothetical protein |
| M20 | SPV_1100 **C30A** F10L  SPV_1211 **C457T** D153N | Glucose-6-phosphate 1-dehydrogenase ***zwf***  3-dehydroquinate dehydratase I ***aroD*** |
| M21 | SPV_1211 **C457T** D153N | 3-dehydroquinate dehydratase I ***aroD*** |
| M23 | SPV_0018 **G2633A** R878K  SPV_0319 **C772T** H258Y  SPV_1211 **C457T** D153N | 23S ribosomal RNA ***rrlA***  Undecaprenyl-phosphate galactosephosphotransferase ***cps2E***  3-dehydroquinate dehydratase I ***aroD*** |
| M36 | SPV_0328 **G445T** D149Y | Glucose-1-phosphate thymidylyltransferase ***cps2L*** |
| M37 | SPV_0444 **C452T** P151L  SPV_1672 **G746A** S249F  SPV_1679 **A140C** Q47P  SPV_1987 **C691T** D231N | Endo-beta-N-acetylglucosaminidase ***endoD***  Wall teichoic acid biosynthesis protein ***tacL***  Multiple sugar metabolism operon regulatory protein ***msmR/rafR***  Fucolectin-related protein ***fucL*** |
| M39 | SPV_1100 **G43A** D15N | Glucose-6-phosphate 1-dehydrogenase ***zwf*** |
| **Negative Transformation clones^d^** | | |
| N40 | SPV_0773 **T1217G** V406G | Fructose-specific PTS IIABC components ***fruA*** |
| N41 | SPV_0152 **C1000T** R334C  SPV_1401 **C93A** L31F  SPV_1676 **G754C** L244V | N-acyl-L-amino acid amidohydrolase ***dapE***  Dihydrofolate reductase ***folA***  Multiple sugar ABC transporter, membrane-spanning permease protein ***msmF/rafF*** |
| N42 | SPV_0080 **G3687A** M1229I  SPV_1100 **G1243A** E415K | Cell wall surface anchor family protein ***pavB/pfbB***  Glucose-6-phosphate 1-dehydrogenase ***zwf*** |
| N43 | SPV_0444 **C452T** P151L  SPV_1672 **G746A** S249F  SPV_1987 **C691T** D231N | Endo-beta-N-acetylglucosaminidase ***endoD***  Wall teichoic acid biosynthesis protein ***tacL***  Fucolectin-related protein ***fucL*** |
| N44 | SPV_0237 **C326T** P109L  SPV_0277 **G659A** R220Q  SPV_0346 **G349A** E117K  SPV_0538 **G1618A** D540N  SPV_0700 **G868A** V290M  SPV_0760 **G896A** G299E  SPV_0852 **C188T** A63V  SPV_1004 **G104A** A35V  SPV_1104 **G1871A** T624M  SPV_1130 **G150A** W50*  SPV_1168 **C818T** G273E  SPV_1401 **C93A** L31F  SPV_1541 **C319T** A107T  SPV_1671 **GC1516G^b^**  SPV_1676 **C624T** W208*  SPV_1902 **C1486T** D496N  SPV_1903 **C853T** A285T  SPV_2013 **C677T** G226D | Glycerol dehydrogenase ***gldA***  6-phospho-beta-glucosidase ***celA***  Mevalonate kinase ***mvk***  Excinuclease ABC subunit C ***uvrC***  Lysyl aminopeptidase ***pepN***  DNA polymerase III subunits gamma and tau ***dnaX***  Dihydroorotate dehydrogenase (NAD(+)), catalytic subunit ***pyrD***  Glyceraldehyde-3-phosphate dehydrogenase (NADP(+)) ***gapN***  Chromosome partition protein ***smc***  Lipopolysaccharide cholinephosphotransferase ***licD***  Dipeptide transport system permease protein ***appC***  Dihydrofolate reductase ***folA***  Hypothetical protein  Peptide ABC transporter substrate-binding protein ***amiA***  Multiple sugar ABC transporter, membrane-spanning permease protein ***msmF/rafF***  Multidrug resistance ABC transporter ATP-binding and permease protein ***patA***  DNA mismatch repair protein ***hexA/mutS***  Glycerol kinase ***glpK*** |
| N46 | SPV_1667 **C832T** E278K  SPV_1671 **C1591T** G531R  SPV_1675 **G101T** S34Y | Peptide ABC transporter ATP-binding protein ***amiF***  Peptide ABC transporter substrate-binding protein ***amiA***  Multiple sugar ABC transporter, membrane-spanning permease protein ***msmG/rafG*** |
| N47 | SPV_0492 **C394T** H132Y  SPV_0561 **G883A** G295S  SPV_0593 **G97A** E33K  SPV_1018 **G1597A** P533S  SPV_1337 **C1207T** D403N  SPV_2016 **G409A** D127N | Hypothetical protein  Hypothetical protein  GTP-binding protein *t****ypA/bipA***  IgA1 protease ***zmpA***  ATP synthase F1 sector alpha chain ***atpA***  tRNA dihydrouridine synthase B ***dusB*** |
| N48 | SPV_0328 **G430T** G144C  SPV_0623 **G181A** G61S  SPV_1211 **C457T** D153N  SPV_1676 **C467A** G148V | Glucose-1-phosphate thymidylyltransferase ***cps2L***  Hydroxyethylthiazole kinase ***thiM1***  3-dehydroquinate dehydratase I ***aroD***  Multiple sugar ABC transporter, membrane-spanning permease protein ***msmF/rafF*** |
| N49 | SPV_0018 **G2633A** R878K  SPV_0319 **C665T** T222I  SPV_1211 **C457T** D153N | 23S ribosomal RNA ***rrlA***  Undecaprenyl-phosphate galactosephosphotransferase ***cps2E***  3-dehydroquinate dehydratase I ***aroD*** |
| N50 | SPV_1667 **C832T** E278K  SPV_1671 **C1591T** G531R  SPV_1675 **G101T** S34Y | Peptide ABC transporter ATP-binding protein ***amiF***  Peptide ABC transporter substrate-binding protein ***amiA***  Multiple sugar ABC transporter, membrane-spanning permease protein ***msmF/rafF*** |

^a^ Asterisks denotes non-sense mutations. DNA mutations are shown in bold.

^b^ Indels leading to frameshifts

^c^ These cells have in principle acquired the capacity for natural transformation in C+Y but did not have the expected FolA^I100L^ genotype and were excluded from detailed analysis. FolA mutations selected de novo in these mutants and explaining their trimethoprim resistance is shown in blue. Twenty clones were sequenced but M9 had only mutations in intergenic regions (see Table S5) and we could not observe any mutations in M8.

^d^ Trimethoprim resistant colonies that did not lead to streptomycin-resistant transformants in the secondary screen (Fig.S3). Eleven clones were sequenced but no mutations were found in N45.
