# Supplementary material for: Point mutations in functionally diverse genes are associated with increased natural DNA transformation in multidrug resistant Streptococcus pneumoniae
Source: Nucleic Acids Res. 2024 Dec 3;53(1):gkae1140. doi: 10.1093/nar/gkae1140 (PMC11724299; doi:10.1093/nar/gkae1140)
Supplement: gkae1140_Supplemental_Files [file gkae1140_supplemental_files.zip › Table S5.docx]

**Table S5. Mutations in intergenic regions among EMS treated *S. pneumoniae* D39V clones with increased natural transformation in C+Y medium.**

| **Mutant** | **Mutations^a^** | **Gene name** |
| --- | --- | --- |
| **Hyper-transformants with increased natural transformation with the FolA^I100L^ genotype** | | |
| T3 | intergenic_SPV_1382_SPV_1383  (C1401731T) | glutathione-dependent disulfide-bond oxidoreductase (SPV_1382); cation-translocating P-type ATPase *caxP* (SPV_1383) |
| T18 | Intergenic_SPV_1382_SPV_1383  (C1401731T) | glutathione-dependent disulfide-bond oxidoreductase (SPV_1382); cation-translocating P-type ATPase *caxP* (SPV_1383) |
| **Mutants with increased natural transformation but without the FolA^I100L^ genotype** | | |
| M2 | intergenic_SPV_0717_SPV_0718  (T734227A) | ATP-dependent Clp protease ATP-binding subunit *clpE* (SPV_0717); DUF1797 family protein (SPV_0718) |
| M2 | Intergenic_SPV_1401_SPV_1402  (C1421442T) | Dihydrofolate reductase *folA* (SPV_1401); Bifunctional non-specific DNA-binding protein/ferroxidase *dpr* (SPV_1402) |
| M4 | intergenic_SPV_2027_SPV_2028  (C2006870T) | hypothetical protein (SPV_2027);  [Choline binding protein D *cbp3*](https://www.patricbrc.org/view/Feature/PATRIC.1313.15790.CP027540.CDS.2006969.2008315.rev) (SPV_2028) |
| M4 | Intergenic_SPV_1401_SPV_1402  (C1421442T) | Dihydrofolate reductase *folA* (SPV_1401); Bifunctional non-specific DNA-binding protein/ferroxidase *dpr* (SPV_1402) |
| M5 | Intergenic_SPV_0768_SPV_0769  (T781355C) | AI-2E family transporter *cozE* (SPV_0768); transfer-messenger RNA (SPV_0769) |
| M5 | Intergenic_SPV_1401_SPV_1402  (C1421442T) | Dihydrofolate reductase *folA* (SPV_1401); Bifunctional non-specific DNA-binding protein/ferroxidase *dpr* (SPV_1402) |
| M5 | intergenic_SPV_2027_SPV_2028  (C2006870T) | hypothetical protein (SPV_2027);  [Choline binding protein D *cbp3*](https://www.patricbrc.org/view/Feature/PATRIC.1313.15790.CP027540.CDS.2006969.2008315.rev) (SPV_2028) |
| M6 | Intergenic_SPV_1401_SPV_1402  (C1421459A) | Dihydrofolate reductase *folA* (SPV_1401); Bifunctional non-specific DNA-binding protein/ferroxidase *dpr* (SPV_1402) |
| M7 | Intergenic_SPV_0525_SPV_0526  (G537300A) | Sensor histidine kinase *vncS* (SPV_0525); fructose-biphosphate aldolase *fba* (SPV_0526) |
| M7 | Intergenic_SPV_1401_SPV_1402  (C1421459T) | Dihydrofolate reductase *folA* (SPV_1401); Bifunctional non-specific DNA-binding protein/ferroxidase *dpr* (SPV_1402) |
| M8 | Intergenic_SPV_1401_SPV_1402  (C1421459T) | Dihydrofolate reductase *folA* (SPV_1401); Bifunctional non-specific DNA-binding protein/ferroxidase *dpr* (SPV_1402) |
| M9 | Intergenic_SPV_1460_SPV_1461  (C1476811A) | Endopeptidase *pepO* (SPV_1460); metal ABC transporter ATP-binding protein *psaB* (SPV_1461) |
| M9 | Intergenic_SPV_1902_SPV_1903  (C1873762A) | ABC transporter ATP-binding protein *patA* (SPV_1902); DNA mismatch repair protein *mutS* (SPV_1903) |
| M9 | Intergenic_SPV_1401_SPV_1402  (C1421459T) | Dihydrofolate reductase *folA* (SPV_1401); Bifunctional non-specific DNA-binding protein/ferroxidase *dpr* (SPV_1402) |
| M10 | Intergenic_SPV_1401_SPV_1402  (C1421459T) | Dihydrofolate reductase *folA* (SPV_1401); Bifunctional non-specific DNA-binding protein/ferroxidase *dpr* (SPV_1402) |
| M11 | Intergenic_SPV_1401_SPV_1402  (C1421459T) | Dihydrofolate reductase *folA* (SPV_1401); Bifunctional non-specific DNA-binding protein/ferroxidase *dpr* (SPV_1402) |
| M12 | Intergenic_SPV_1401_SPV_1402  (C1421459T) | Dihydrofolate reductase *folA* (SPV_1401); Bifunctional non-specific DNA-binding protein/ferroxidase *dpr* (SPV_1402) |
| M13 | Intergenic_SPV_1401_SPV_1402  (C1421459T) | Dihydrofolate reductase *folA* (SPV_1401); Bifunctional non-specific DNA-binding protein/ferroxidase *dpr* (SPV_1402) |
| M17 | Intergenic_SPV_1401_SPV_1402  (C1421442T) | Dihydrofolate reductase *folA* (SPV_1401); Bifunctional non-specific DNA-binding protein/ferroxidase *dpr* (SPV_1402) |
| M19 | Intergenic_SPV_1505_SPV_1506  (G1526528A) | Hypothetical protein (SPV_1505); axetylxylan esterase *axe1* (SPV_1506) |
| M19 | Intergenic_SPV_1629_SPV_1630  (G1645165A) | Purine permease *pbuX* (SPV_1629); protein *dpnD* (SPV_1630) |
| M19 | Intergenic_SPV_1745_SPV_1746  (C1739654CA) | XRE family transcriptional regulator *plcR* (SPV_1745); Hypothetical protein (SPV_1746) |
| M19 | Intergenic_SPV_1401_SPV_1402  (C1421442T) | Dihydrofolate reductase *folA* (SPV_1401); Bifunctional non-specific DNA-binding protein/ferroxidase *dpr* (SPV_1402) |
| M20 | Intergenic_SPV_1505_SPV_1506  (G1526528A) | Hypothetical protein (SPV_1505); axetylxylan esterase *axe1* (SPV_1506) |
| M20 | Intergenic_SPV_1629_SPV_1630  (G1645165A) | Purine permease *pbuX* (SPV_1629); protein *dpnD* (SPV_1630) |
| M20 | Intergenic_SPV_1401_SPV_1402  (C1421442T) | Dihydrofolate reductase *folA* (SPV_1401); Bifunctional non-specific DNA-binding protein/ferroxidase *dpr* (SPV_1402) |
| M21 | Intergenic_SPV_1505_SPV_1506  (G1526528A) | Hypothetical protein (SPV_1505); axetylxylan esterase *axe1* (SPV_1506) |
| M21 | Intergenic_SPV_1629_SPV_1630  (G1645165A) | Purine permease *pbuX* (SPV_1629); protein *dpnD* (SPV_1630) |
| M21 | Intergenic_SPV_1902_SPV_1903  (G1873745A) | ABC transporter ATP-binding protein *patA* (SPV_1902); DNA mismatch repair protein *mutS* (SPV_1903) |
| M21 | Intergenic_SPV_1401_SPV_1402  (C1421442T) | Dihydrofolate reductase *folA* (SPV_1401); Bifunctional non-specific DNA-binding protein/ferroxidase *dpr* (SPV_1402) |
| M23 | Intergenic_SPV_1505_SPV_1506  (G1526528A) | Hypothetical protein (SPV_1505); axetylxylan esterase *axe1* (SPV_1506) |
| M23 | Intergenic_SPV_1629_SPV_1630  (G1645165A) | Purine permease *pbuX* (SPV_1629); protein *dpnD* (SPV_1630) |
| M23 | Intergenic_SPV_1401_SPV_1402  (C1421442T) | Dihydrofolate reductase *folA* (SPV_1401); Bifunctional non-specific DNA-binding protein/ferroxidase *dpr* (SPV_1402) |
| M36 | Intergenic_SPV_1902_SPV_1903  (C1873762A) | ABC transporter ATP-binding protein *patA* (SPV_1902); DNA mismatch repair protein *mutS* (SPV_1903) |
| M36 | Intergenic_SPV_1401_SPV_1402  (C1421459T) | Dihydrofolate reductase *folA* (SPV_1401); Bifunctional non-specific DNA-binding protein/ferroxidase *dpr* (SPV_1402) |
| M37 | Intergenic_SPV_1401_SPV_1402  (C1421442T) | Dihydrofolate reductase *folA* (SPV_1401); Bifunctional non-specific DNA-binding protein/ferroxidase *dpr* (SPV_1402) |
| M39 | Intergenic_SPV_1677_SPV_1678  (C1693776A) | sugar ABC transporter substrate-binding protein *rafE* (SPV_1677); alpha-galactosidase *aga* (SPV_1678) |
| M39 | Intergenic_SPV_1401_SPV_1402  (C1421459T) | Dihydrofolate reductase *folA* (SPV_1401); Bifunctional non-specific DNA-binding protein/ferroxidase *dpr* (SPV_1402) |
| **Negative Transformation clones** | | |
| N40 | Intergenic_SPV_2008_SPV_2009  (G1986072T) | Mobile element protein (SPV_2008); hypothetical protein (SPV_2009) |
| N40 | Intergenic_SPV_1401_SPV_1402  (C1421459T) | Dihydrofolate reductase *folA* (SPV_1401); Bifunctional non-specific DNA-binding protein/ferroxidase *dpr* (SPV_1402) |
| N41 | Intergenic_SPV_2218_SPV_0674  (T694423C) | Hypothetical protein (SPV_2218); SSU ribosomal protein S16p *rpsP* (SPV_0674) |
| N41 | Intergenic_SPV_1902_SPV_1903  (G1873745T) | ABC transporter ATP-binding protein *patA* (SPV_1902); DNA mismatch repair protein *mutS* (SPV_1903) |
| N41 | Intergenic_SPV_1401_SPV_1402  (C1421459T) | Dihydrofolate reductase *folA* (SPV_1401); Bifunctional non-specific DNA-binding protein/ferroxidase *dpr* (SPV_1402) |
| N42 | Intergenic_SPV_1401_SPV_1402  (C1421459T) | Dihydrofolate reductase *folA* (SPV_1401); Bifunctional non-specific DNA-binding protein/ferroxidase *dpr* (SPV_1402) |
| N43 | Intergenic_SPV_1401_SPV_1402  (C1421442T) | Dihydrofolate reductase *folA* (SPV_1401); Bifunctional non-specific DNA-binding protein/ferroxidase *dpr* (SPV_1402) |
| N44 | Intergenic_SPV_1929_SPV_1930  (C1901823T) | [23S rRNA (guanine(748)-N(1))-methyltransferase](https://www.patricbrc.org/view/Feature/PATRIC.1313.15790.CP027540.CDS.1900461.1901309.fwd) (SPV_1929); hypothetical protein (SPV_1930) |
| N45 | Intergenic_SPV_1401_SPV_1402  (C1421459T) | Dihydrofolate reductase *folA* (SPV_1401); Bifunctional non-specific DNA-binding protein/ferroxidase *dpr* (SPV_1402) |
| N46 | Intergenic_SPV_1681_SPV_1682  (TTTCAACCCACTACAGTTGACAAAGAGCCAAAAAAGAAAGGACGAAATTTGTCCTTTCTCGAGCTTAGCTTTTC1698570T) | ISL3 family transposase (SPV_1681); tRNA-Ser (SPV_1682) |
| N46 | Intergenic_SPV_1401_SPV_1402  (C1421459T) | Dihydrofolate reductase *folA* (SPV_1401); Bifunctional non-specific DNA-binding protein/ferroxidase *dpr* (SPV_1402) |
| N47 | Intergenic_SPV_1984_SPV_1985  (G1958232A) | SPFH domain-containing protein *ybbK* (SPV_1984);  lactaldehyde reductase *adh2* (SPV_1985) |
| N47 | Intergenic_SPV_1401_SPV_1402  (C1421442T) | Dihydrofolate reductase *folA* (SPV_1401); Bifunctional non-specific DNA-binding protein/ferroxidase *dpr* (SPV_1402) |
| N48 | Intergenic_SPV_1505_SPV_1506  (G1526528A) | Hypothetical protein (SPV_1505); axetylxylan esterase *axe1* (SPV_1506) |
| N48 | Intergenic_SPV_1629_SPV_1630  (G1645165A) | Purine permease *pbuX* (SPV_1629); protein *dpnD* (SPV_1630) |
| N48 | Intergenic_SPV_1401_SPV_1402  (C1421442T) | Dihydrofolate reductase *folA* (SPV_1401); Bifunctional non-specific DNA-binding protein/ferroxidase *dpr* (SPV_1402) |
| N49 | Intergenic_SPV_1505_SPV_1506 (G1526528A) | Hypothetical protein (SPV_1505); axetylxylan esterase *axe1* (SPV_1506) |
| N49 | Intergenic_SPV_1629_SPV_1630 (G1645165A) | Purine permease *pbuX* (SPV_1629); protein *dpnD* (SPV_1630) |
| N49 | Intergenic_SPV_1401_SPV_1402  (C1421442T) | Dihydrofolate reductase *folA* (SPV_1401); Bifunctional non-specific DNA-binding protein/ferroxidase *dpr* (SPV_1402) |
| N50 | Intergenic_SPV_1069_SPV_1070 (G1094475A) | Bacteriocin immunity protein (SPV_1069);  tRNA pseudouridine(55) synthase *truB* (SPV_1070) |
| M50 | Intergenic_SPV_1401_SPV_1402  (C1421459T) | Dihydrofolate reductase *folA* (SPV_1401); Bifunctional non-specific DNA-binding protein/ferroxidase *dpr* (SPV_1402) |

In blue, mutations explaining trimethoprim resistance in FolA^I100L-^ genotype mutants.

^a^ For mutations in intergenic regions, the chromosomal position with the mutation is indicated within parentheses.
